# Surface bio-erosion and structural alteration of colored and agricultural plastics induced by *Scenedesmus dimorphus* and *Chlorella vulgaris*

**DOI:** 10.3389/fmicb.2026.1934915

**Published:** 2026-09-23

**Authors:** Aysu Besler, Özlem Gürünlü Alma, Murat Can Sunar

**Affiliations:** 1 Department of Biology, Faculty of Science, Muğla Sıtkı Koçman University, Muğla, Türkiye; 2 Department of Statistics, Faculty of Science, Muğla Sıtkı Koçman University, Muğla, Türkiye; 3 Faculty of Fisheries, Muğla Sıtkı Koçman University, Muğla, Türkiye

**Keywords:** bioremediation, *Chlorella vulgaris*, commercial plastic formulations, low density polyethylene, microalgal mediated plastic alteration, polyethylene terephthalate, *Scenedesmus dimorphus*

## Abstract

Plastic pollution remains a major environmental challenge because synthetic polymers and their additives can resist biological degradation. This study investigated the early stage surface modification of commercially formulated plastics following exposure to the freshwater microalgae *Scenedesmus dimorphus* and *Chlorella vulgaris*. Transparent and pigmented polyethylene terephthalate (PET) bottles and agricultural low density polyethylene (LDPE) films were incubated with microalgal cultures for 100 days under controlled laboratory conditions. Changes in plastic mass and surface properties were evaluated using gravimetric measurements, Fourier-transform infrared spectroscopy (FTIR), and scanning electron microscopy (SEM). Weight loss varied among treatment and plastic combinations. Aligned Rank Transform analysis showed significant effects of treatment (*F* = 22.54, *p* < 0.001), plastic type (*F* = 2.50, *p* = 0.036), and their interaction (*F* = 3.81, *p* < 0.001). FTIR analysis indicated changes in carbonyl and aliphatic functional groups, consistent with oxidative surface modification. SEM observations showed surface roughening, cracks, cavities, and localized delamination after microalgal exposure. The magnitude of these changes varied among plastic formulations and microalgal treatments. The results indicate that *S. dimorphus* and *C. vulgaris* were associated with detectable physicochemical and morphological changes at the plastic surface during the 100 day incubation period. However, the observed changes represent early stage surface modification and do not demonstrate complete polymer biodegradation or mineralization. The findings highlight the importance of polymer formulation when evaluating microalgae plastic interactions and provide evidence for further investigation of microalgal surface associated plastic alteration.

## 1 Introduction

Plastic pollution is a major environmental challenge because of the extensive production, persistence, and accumulation of synthetic polymers in terrestrial and aquatic ecosystems. Conventional plastics such as polyethylene terephthalate (PET), polyethylene (PE), and polypropylene (PP) possess chemically stable structures that limit natural degradation and contribute to long term environmental persistence (Shah et al., 2008; Tokiwa et al., 2009). Their widespread use in packaging, agriculture, fisheries, and consumer products has further increased plastic waste and microplastic contamination worldwide (Ogunola and Palanisami, 2016). Although physical and chemical recycling technologies are available, their application remains constrained by energy requirements, heterogeneous waste streams, and limited recycling efficiency (Danso et al., 2019).

The environmental behavior of commercial plastics is also influenced by additives, including pigments, plasticizers, UV stabilizers, antioxidants, and other formulation components. These compounds can modify polymer physicochemical properties and surface characteristics and may influence microbial attachment and polymer transformation (Wei and Zimmermann, 2017; Cai et al., 2023). Pigments primarily provide color but may also modify the susceptibility of polymer surfaces to photochemical alteration by absorbing UV radiation (Zhao et al., 2022). Thus, commercially formulated plastics may exhibit responses to environmental and biological processes that differ from those of additive free polymers.

Low density polyethylene (LDPE) is extensively used in agriculture, particularly for greenhouse coverings and mulch films because of its low cost, flexibility, durability, and favorable light transmission properties. Agricultural plastic use has increased substantially, with mulch films covering more than 128,000 km^2^ worldwide (Dilara and Briassoulis, 2000; Lin and Zhang, 2025). During use, LDPE films are exposed to UV radiation, temperature fluctuations, agricultural chemicals, and mechanical stress, promoting surface deterioration and fragmentation and contributing to plastic accumulation in agricultural soils (Huang et al., 2020; Zhou et al., 2020; Liu et al., 2021; Gao et al., 2021; Chen et al., 2020). Such residues can alter soil structure, water retention, hydraulic conductivity, nutrient cycling, microbial communities, and plant growth (de Souza Machado et al., 2018; Huang et al., 2019). Their persistence has therefore stimulated research into biological processes capable of colonizing, modifying, or transforming polyethylene based materials (Kaushal et al., 2021; Yao et al., 2022; Yuan et al., 2022; Rong et al., 2024; Campanale et al., 2024).

Microbial approaches have therefore attracted increasing attention as potential alternatives or complementary strategies for plastic waste management (Ganesh Kumar et al., 2020). Bacteria and fungi capable of degrading synthetic polymers through extracellular enzymatic activity have been widely investigated (Mohanan et al., 2020; Roy and Chakraborty, 2024; Purohit et al., 2025; Ali et al., 2021; Fortaleza et al., 2026; Khan et al., 2017). In contrast, microalgal interactions with plastics remain less well characterized. Microalgae can form biofilms and produce extracellular polymeric substances (EPS), extracellular metabolites, and reactive oxygen species that may influence polymer surface properties (Brasil et al., 2017; Hadiyanto et al., 2022; Feisal et al., 2025; Kapelewska et al., 2025; Sarkhel et al., 2026). However, current evidence indicates that microalgal interactions with plastics involve processes such as surface colonization, aggregation, EPS mediated attachment, and oxidative modification, while their contribution to actual polymer degradation remains uncertain (Feisal et al., 2025).

Recent studies further indicate that microplastic characteristics influence microalgal responses. A review focusing on *Chlorella vulgaris* reported that plastic type, surface properties, aggregation, aging, and chemical leaching can affect algal growth, photosynthetic activity, and oxidative stress (Acharjee et al., 2026). Species specific physiological differences may also influence microalgal responses to environmental stressors, emphasizing the importance of evaluating plastic algal interactions at the species level (Nur et al., 2026). These findings support the need to consider both polymer formulation and microalgal characteristics when assessing plastic surface interactions.

Microalgae may interact with plastic surfaces through biofilm formation, EPS production, surface attachment, oxidative modification, and interactions with associated microbial communities (Hadiyanto et al., 2022). In freshwater systems, *Scenedesmus dimorphus* has been reported to form biofilms and induce detectable changes in LDPE, including approximately 3.7% mass loss under specific experimental conditions (Jiao et al., 2026). *Scenedesmus* and *Chlorella* species have also demonstrated interactions with plastic particles under contaminated aquatic conditions (Zhao et al., 2026). Nevertheless, changes in mass, morphology, or functional groups should not be interpreted alone as evidence of complete polymer biodegradation.

A major methodological challenge in plastic biodegradation research is distinguishing biological degradation from surface modification, weathering, fragmentation, and biofouling (Montazer et al., 2020). Surface oxidation and physical deterioration may occur without substantial conversion of polymer carbon into CO₂, biomass, or other mineralization products. Accordingly, gravimetric measurements, FTIR, and SEM provide complementary evidence of changes in mass, surface chemistry, and morphology but cannot independently establish complete biodegradation or mineralization (Mohanan et al., 2020). This distinction is particularly important when evaluating commercially formulated plastics, for which changes in pigments and additives may further influence surface responses.

In this study, we investigated the interactions of two freshwater microalgae, *Scenedesmus dimorphus* and *Chlorella vulgaris*, with commercially available PET and LDPE plastics containing different formulations. Gravimetric measurements, FTIR spectroscopy, and SEM observations were combined to evaluate changes in plastic mass, surface functional groups, and morphology following microalgal exposure. Particular attention was given to differences among transparent and pigmented PET materials and agricultural LDPE formulations. We hypothesized that differences in polymer formulation would be associated with different degrees of microalgal associated surface alteration.

The study was designed to characterize early stage physicochemical and morphological changes at plastic surfaces, rather than to demonstrate complete polymer biodegradation or mineralization. By focusing on formulation dependent surface responses, this work aims to clarify the initial interactions between freshwater microalgae and commercially formulated plastics and provide a more cautious basis for evaluating their potential role in biological plastic remediation strategies.

## 2 Materials and methods

### 2.1 Microalgal cultures and culture conditions

*Scenedesmus dimorphus* (strain T01) and *Chlorella vulgaris* were obtained from the MCS Aquatic R&D Aquaculture Culture Collection and used throughout the study.

Both strains were cultivated in Bold’s Basal Medium (BBM), prepared according to the standard formulation described by Stein (1973). BBM was prepared from sterile stock solutions containing sodium nitrate (NaNO₃), calcium chloride dihydrate (CaCl₂·2H₂O), magnesium sulfate heptahydrate (MgSO₄·7H₂O), dipotassium hydrogen phosphate (K₂HPO₄), potassium dihydrogen phosphate (KH₂PO₄), sodium chloride (NaCl), PIV trace metal solution, and vitamin solution. For each liter of distilled water, 5 mL NaNO₃ stock solution, 1 mL CaCl₂·2H₂O stock solution, 1 mL MgSO₄·7H₂O stock solution, 1 mL K₂HPO₄ stock solution, 2 mL KH₂PO₄ stock solution, 1 mL NaCl stock solution, 6 mL PIV trace element solution, and 1 mL vitamin solution were added. The culture medium was sterilized by filtration through a sterile 0.2 μm membrane filter before inoculation (Stein, 1973).

Microalgal cultures were inoculated at an initial cell density of 1 × 10^6^ cells mL^−1^ in sterile BBM. Cultures were maintained at 23 ± 1 °C, with the culture pH maintained between 7.0 and 8.0 throughout the experimental period. A 12:12 h light:dark photoperiod was applied at a constant light intensity of 40 μmol photons m^−2^ s^−1^, which supports stable cultivation of freshwater green microalgae (Bialevich et al., 2022; Gao et al., 2022). Cultures were gently mixed by manual swirling twice daily to maintain homogeneous cell suspension and facilitate gas exchange.

### 2.2 Preparation of plastic samples

Plastic substrates were selected to represent post consumer polyethylene terephthalate (PET) packaging and agricultural low density polyethylene (LDPE) films with different commercial formulations. The materials included gray, blue, and green pigmented PET beverage bottles; transparent PET drinking water bottles; white pigmented LDPE greenhouse covering film (Sepas, Turkey; 24-month UV-stabilized material containing an EVA additive); and black pigmented LDPE mulch film (İleri Plastik, Turkey; 12 M-B ECO grade). These materials were selected because PET and LDPE are widely used in packaging and agricultural applications and exhibit high environmental persistence (Shah et al., 2008; Wei and Zimmermann, 2017; Dilara and Briassoulis, 2000). Consumer PET bottles were obtained from local retail markets, whereas the agricultural LDPE materials were supplied directly by the respective manufacturers. The materials were therefore treated as commercial plastic formulations, and differences among colors or formulations were not interpreted as effects of individual pigments or additives because the chemical identity and concentration of individual formulation components were not independently determined. All materials were cut into 1 × 1 cm fragments using sterile scissors. These specimens were referred to throughout the study as plastic fragments rather than microplastics because their dimensions exceeded the commonly used 5 mm size threshold for microplastic particles.

Before incubation, the plastic fragments were surface sterilized by immersion in 70% (v/v) ethanol for 30 min, following a procedure previously used for polyethylene in biodegradation experiments (Afify et al., 2025). The fragments were subsequently rinsed with sterile distilled water for 5 min to remove residual ethanol, air dried under aseptic conditions, and stored in sterile containers until experimental use.

### 2.3 Experimental design and incubation conditions

The experiments were designed as a two-factor factorial experiment with microalgal treatment and plastic formulation as the experimental factors. The treatment factor consisted of three levels:

1. Control,
2. *Scenedesmus dimorphus*, and
3. *Chlorella vulgaris*.

The plastic factor consisted of six levels:

1. Blue PET,
2. Green PET,
3. Gray PET,
4. Black LDPE,
5. White LDPE, and
6. Transparent PET.

Thus, the experimental design comprised 3 treatment groups × 6 plastic types = 18 experimental combinations. Six independent biological replicates were prepared for each combination (n = 6), resulting in 108 biological experimental units.

Each biological replicate consisted of a sterile 250 mL Erlenmeyer flask containing 160 mL of actively growing microalgal culture and four plastic fragments measuring 1 × 1 cm each. The four fragments within each flask were considered technical replicates and were pooled for gravimetric analysis. Consequently, the individual flask/biological replicate, rather than each plastic fragment, was considered the experimental unit for statistical analysis.

Control flasks contained the same plastic materials in sterile BBM without microalgal inoculation and were incubated under otherwise identical conditions. These controls were included to distinguish changes associated with microalgal exposure from changes occurring in the absence of microalgal inoculation. The incubation period was 100 days under the culture conditions described in Section 2.1.

### 2.4 Gravimetric analysis

Following the 100 day incubation period, the plastic fragments were recovered and subjected to the standardized cleaning procedure described in Section 2.5. After cleaning, the samples were dried to constant weight before gravimetric measurements. To minimize analytical variability associated with the low mass of individual plastic fragments, the four fragments belonging to each biological replicate were weighed collectively using a Sartorius balance (Sartorius, Germany) with a readability of 0.0001 g. The combined initial mass of the four fragments was recorded before incubation as W₀, whereas the combined final mass after incubation and cleaning was recorded as W₁. Weight loss (WL) was calculated according to Equation 1 (Taghavi et al., 2021):

$$\mathrm{WL}(\text{Weight loss}\%)=(\frac{W_{0}-W_{1}}{W_{0}})\times 100$$ (1)

Where W₀ represents the initial dry mass of the pooled plastic fragments before incubation and W₁ represents the final dry mass after incubation and post treatment cleaning.

The calculated WL(%) value for each flask was treated as one independent biological observation. The four fragments within each flask were therefore considered technical replicates and were not treated as independent observations in the statistical analysis. This approach was adopted to avoid pseudoreplication and to ensure that the experimental unit corresponded to the independent biological replicate.

Negative weight loss values were retained in the dataset because they represent cases in which the measured final mass exceeded the initial mass. Such values may result from analytical variability, incomplete removal of surface associated material, or other experimental sources of variation and were therefore not excluded solely because they were negative. Similarly, unusually high observations were retained in the primary analysis and evaluated through a separate sensitivity analysis rather than being removed solely on the basis of their magnitude.

Gravimetric weight loss was used as an indicator of net mass change in the plastic specimens. Because mass loss alone cannot distinguish polymer biodegradation from physical fragmentation, surface associated material removal, or other physicochemical processes, gravimetric results were interpreted together with FTIR and SEM observations rather than as independent evidence of complete polymer biodegradation or mineralization (Mohanan et al., 2020).

### 2.5 Post-incubation cleaning and sample preparation

Following incubation, all plastic fragments were subjected to a standardized cleaning procedure before gravimetric measurements and surface characterization by scanning electron microscopy (SEM) and Fourier-transform infrared spectroscopy (FTIR). The purpose of this procedure was to remove attached algal biomass, loosely associated organic material, and inorganic deposits from the plastic surfaces before characterization.

The fragments were first immersed in 2% (w/v) sodium dodecyl sulfate (SDS) solution for 30 min and subsequently rinsed thoroughly with deionized water. The samples were then immersed in 0.1 M hydrochloric acid (HCl) for 15 min to remove inorganic deposits and were subsequently rinsed again with deionized water.

When persistent organic or biological residues remained on the plastic surfaces after the SDS and HCl treatments, the samples were additionally treated with 30% hydrogen peroxide (H₂O₂) at room temperature for 30 min to remove residual organic material and attached biomass. Following the H₂O₂ treatment, the plastic fragments were thoroughly rinsed with deionized water and subsequently dried in a laboratory oven at 50–60 °C to constant weight. The samples were then cooled to room temperature in a desiccator and weighed before subsequent FTIR and SEM analyses. The same cleaning procedure was applied to both microalgae exposed and control samples to ensure consistent removal of residual biomass and organic material prior to gravimetric and surface characterization analyses (Mohanan et al., 2020).

To ensure that the washing protocol did not artifactually alter the plastic surfaces, abiotic controls (plastics incubated in algae free medium) were subjected to identical 30%H₂O₂ cleaning at room temperature. No significant mass variations (∆m < 0.1%) or FTIR chemical alterations (e.g., unexpected carbonyl band formation) were observed in abiotic controls post cleaning.

### 2.6 Fourier transform infrared spectroscopy (FTIR-ATR) analysis

Fourier transform infrared spectroscopy with attenuated total reflectance (FTIR-ATR) was used to characterize changes in the surface chemical structure and functional groups of the plastic fragments before and after the 100 day incubation period. Measurements were performed using a Thermo Scientific Nicolet iS10 Fourier transform infrared spectrometer equipped with a Smart iTR™ attenuated total reflectance accessory with a single reflection diamond crystal.

Before FTIR analysis, plastic fragments were subjected to the post incubation cleaning procedure described in Section 2.5 and dried to constant weight. Both control and microalgae exposed samples were analyzed using the same procedure. The cleaned plastic surface was placed directly in contact with the ATR diamond crystal, and no additional chemical treatment or sample preparation was applied.

Spectra were collected in transmittance mode (%T) over the spectral range of 4,000 to 600 cm^−1^, using a spectral resolution of 4 cm^−1^ and 32 scans per spectrum. An ambient air background spectrum was collected before each measurement to account for atmospheric contributions, particularly carbon dioxide and water vapor. Spectral acquisition and baseline correction were performed using OMNIC™ software (Thermo Fisher Scientific).

The FTIR spectra were evaluated by comparing the characteristic absorption bands of the initial, control, and microalgae exposed plastic samples. For LDPE, the analysis focused on the characteristic bands associated with methylene groups, including C-H stretching, CH₂ bending, and CH₂ rocking vibrations. Changes in bands associated with carbonyl, hydroxyl, and C-O-containing groups were also examined because these functional groups may be associated with oxidative modification of polyethylene surfaces. Characteristic LDPE absorption bands in the regions of approximately 2,915, 2,847, 1,472–1,461, and 730–718 cm^−1^ have been reported in previous FTIR based studies of plastic materials (Oliveira et al., 2022).

For PET, the evaluation focused on characteristic bands associated with ester carbonyl groups, aromatic C-H vibrations, and C-O stretching. Particular attention was given to absorption bands in the regions of approximately 1,715, 1,245, 1,100, 870, and 730 cm^−1^, which have been used for the characterization of PET surface chemical changes (Ioakeimidis et al., 2016).

FTIR spectra were used to identify differences in functional group profiles and relative band intensities among the initial, control, and microalgae exposed samples. FTIR observations were interpreted as evidence of changes in surface chemistry rather than as direct evidence of complete polymer biodegradation. This distinction is important because FTIR detected changes in carbonyl, hydroxyl, or other oxygen containing groups may result from biological or abiotic oxidation, while residual biomass can also contribute to infrared signals if the plastic surface is not adequately cleaned (Sandt et al., 2021). Therefore, FTIR results were evaluated together with gravimetric measurements and SEM observations to characterize early stage surface modification rather than to demonstrate complete polymer degradation or mineralization.

### 2.7 Scanning electron microscopy (SEM) analysis

Scanning electron microscopy (SEM) was used to characterize morphological changes on plastic surfaces after the 100 day incubation period. Following the standardized cleaning and drying procedure described in Section 2.5, both microalgae exposed and control samples were prepared using the same procedure. Surface morphology was examined using a ZEISS EVO 15 scanning electron microscope equipped with a lanthanum hexaboride (LaB₆) electron source. Samples were dehydrated through a graded ethanol series and sputter coated with a thin conductive metal layer before imaging.

Micrographs were acquired at an accelerating voltage of 15.00 kV and working distances of 8.1–9.5 mm. To capture both microscale surface features and finer structural alterations, images were obtained at magnifications ranging from 287 × to 9,570×, with scale bars of 1–20 μm provided in the respective micrographs. Samples exhibiting pronounced gravimetric and FTIR changes were prioritized for higher resolution imaging to facilitate detailed examination of associated surface features. Representative micrographs were selected from examined surface regions showing characteristic morphological features, including microcracks, cavities, surface roughening, delamination, and localized surface irregularities that were consistently observed across the inspected areas.

Because ethanol dehydration and subsequent drying may introduce or accentuate morphological artifacts in polymer surfaces, particularly in thin LDPE films, SEM features were interpreted cautiously. Such features were therefore not considered independent evidence of biological erosion or polymer degradation. The same sample preparation and imaging procedures were applied to control and microalgae exposed specimens to facilitate comparative interpretation. SEM observations were evaluated together with gravimetric and FTIR measurements to provide complementary evidence of surface alteration, while recognizing that morphological changes alone do not establish complete polymer biodegradation or mineralization (Mohanan et al., 2020; Cai et al., 2023).

### 2.8 Statistical analysis

All statistical analyses were conducted using the ARTool, emmeans, car, and rstatix packages in R version 4.3.3 (Fox and Weisberg, 2019; Kassambara, 2023; Kay et al., 2021; Lenth, 2024; R Core Team, 2025). The analysis was based on the two-factor factorial design described in Section 2.3, comprising three microalgal treatment levels (Control, *Scenedesmus dimorphus*, and *Chlorella vulgaris*) and six plastic formulations (Blue PET, Green PET, Gray PET, Black LDPE, White LDPE, and Transparent PET), with six biological replicates per Treatment × Plastic combination (18 combinations; *N* = 108). The Erlenmeyer flask was considered the experimental unit. The four plastic fragments within each flask were treated as technical replicates and were pooled for gravimetric analysis; therefore, they were not treated as independent observations in the statistical analysis.

Weight loss (%) was used as the response variable. Descriptive statistics, including the mean, median, standard deviation (SD), standard error (SE), minimum, and maximum, were calculated for each Treatment × Plastic combination. The distribution of the response variable was assessed within each experimental cell using the Shapiro–Wilk test. Homogeneity of variance was evaluated using the Brown–Forsythe modification of Levene’s test, with the median used as the center of the absolute deviations. A conventional two-way ANOVA was additionally fitted for diagnostic purposes but was not used as the primary inferential analysis.

Because the data showed departures from the assumptions of conventional parametric factorial ANOVA, the primary inferential analysis was performed using the aligned rank transform (ART) procedure implemented in the ARTool package. The ART model included Treatment, Plastic, and the Treatment × Plastic interaction as fixed effects. Main effects and the interaction were evaluated using a two-sided significance level of α = 0.05. Partial eta-squared (ηp^2^) was calculated for each model term as an effect size measure according to $\eta_{p}^{2}=SS_{\text{effect}}/(SS_{\text{effect}}+SS_{\text{error}})$ .

Following the factorial analysis, pairwise comparisons among the 18 Treatment × Plastic combinations were performed using ART-C contrasts with Tukey adjustment for multiple comparisons. All 153 possible pairwise comparisons among the experimental combinations were evaluated. For each contrast, the estimated difference, test statistic, degrees of freedom, Tukey-adjusted *p*-value, and 95% confidence interval were obtained. A standardized effect size index (r) was calculated as $|t|/\sqrt{t^{2}+\mathrm{df}}$ .

To further resolve the Treatment × Plastic interaction, difference of differences contrasts were calculated using the interaction contrast procedure of the ART framework. These contrasts evaluated whether the difference between two treatment levels varied across two plastic formulations. The complete set of treatment pair × plastic pair comparisons comprised 45 interaction contrasts. Tukey-adjusted *p*-values, 95% confidence intervals, and the corresponding standardized effect size index (r) were obtained for these contrasts.

Potentially influential observations were evaluated using studentized residuals, leverage, and Cook’s distance from the corresponding factorial linear model. These diagnostics were used to identify observations requiring further evaluation and were not used as automatic criteria for exclusion. Sensitivity analyses were performed by repeating the ART analysis after separately excluding the observed 24.14% value in the Chlorella × Black LDPE combination, the observed 2.22% value in the Control × Transparent PET combination, and both observations simultaneously. The resulting Treatment, Plastic, and Treatment × Plastic interaction results were compared with those obtained from the complete dataset to evaluate the robustness of the statistical findings. The complete dataset was retained as the primary analysis dataset. All statistical tests were two-sided, with *p* < 0.05 considered statistically significant. Tukey-adjusted *p*-values were used for multiple pairwise and interaction comparisons, and 95% confidence intervals were reported for the corresponding contrasts.

## 3 Results

### 3.1 Gravimetric mass change dynamics

Gravimetric mass loss percentage (WL%) was determined after 100 days of incubation across 18 Treatment × Plastic combinations (*n* = 6 biological replicates per group). Descriptive statistics, including mean, standard deviation (SD), median, minimum, maximum, and 95% confidence intervals (95% CI), are summarized in Table 1. In Control groups, mean weight loss remained near zero across most plastic substrate formulations (e.g., Green PET: 0.04 ± 0.04%; Blue PET: −0.06 ± 0.05%), reflecting minimal abiotic background mass change. Control Black LDPE exhibited a mean mass change of −2.08 ± 0.44%.

**Table 1:** Descriptive statistics of gravimetric weight loss percentage (WL%) across commercial plastic fragment formulations (1 × 1 cm) after 100 days of exposure to Control, *Scenedesmus dimorphus*, and *Chlorella vulgaris* treatments (*n* = 6).

Treatment	Plastic formulation	*n*	Mean WL (%)	SD	Median (%)	Min (%)	Max (%)	95% CI (Lower, Upper)
Control	Blue	6	−0.06	0.05	−0.07	−0.12	0.00	(−0.12, −0.01)
	Green	6	0.04	0.04	0.04	0.00	0.07	(−0.01, 0.08)
	Gray	6	−0.09	0.11	−0.08	−0.24	0.00	(−0.20, 0.02)
	Black	6	−2.08	0.44	−2.17	−2.52	−1.52	(−2.54, −1.61)
	White	6	−0.33	0.19	−0.33	−0.58	0.00	(−0.53, −0.13)
	PetWhite	6	−0.16	1.59	−0.04	−2.78	2.22	(−1.82, 1.51)
*Scenedesmus dimorphus*	Blue	6	0.75	1.86	0.00	−0.09	4.55	(−1.20, 2.71)
	Green	6	0.07	0.24	0.07	−0.22	0.44	(−0.18, 0.33)
	Gray	6	−0.19	0.52	0.04	−1.20	0.17	(−0.73, 0.36)
	Black	6	−0.52	0.80	−0.74	−1.60	0.75	(−1.37, 0.32)
	White	6	0.65	1.13	0.39	−0.43	2.84	(−0.53, 1.84)
	PetWhite	6	−0.11	0.16	−0.07	−0.33	0.08	(−0.28, 0.06)
*Chlorella vulgaris*	Blue	6	−0.05	0.21	0.00	−0.35	0.23	(−0.26, 0.17)
	Green	6	−0.10	0.13	−0.07	−0.36	0.00	(−0.24, 0.04)
	Gray	6	0.08	0.15	0.08	−0.16	0.24	(−0.08, 0.24)
	Black	6	1.93	11.06	−1.90	−5.60	24.14	(−9.68, 13.54)
	White	6	−0.09	0.33	0.00	−0.73	0.16	(−0.44, 0.26)
	PetWhite	6	−0.03	0.15	0.01	−0.27	0.13	(−0.19, 0.13)

SD, Standard deviation; CI, Confidence interval (calculated using Student’s *t*-distribution with t_critica_l = 2.571, df = 5). Positive values represent net gravimetric mass loss percentage; negative values indicate net mass retention/gain.

Following microalgal exposure, gravimetric mass changes displayed polymer dependent patterns. In *Scenedesmus dimorphus* treatments, positive mean mass loss was observed for Blue PET (0.75 ± 1.86%; range: −0.09 to 4.55%) and White LDPE (0.65 ± 1.13%; range: −0.43 to 2.84%), whereas other formulations showed values close to zero or slightly negative (e.g., Gray PET: −0.19 ± 0.52%; PetWhite: −0.11 ± 0.16%).

In *Chlorella vulgaris* treatments, Black LDPE mulch film exhibited a mean WL% of 1.93 ± 11.06%, driven by substantial inter replicate dispersion (range: −5.60 to 24.14%; median: −1.90%). Mass loss in all other *C. vulgaris* exposure groups remained close to zero (ranging from −0.10 ± 0.13% in Green PET to 0.08 ± 0.15% in Gray PET).

Because mean mass loss values in several groups were close to zero or negative, relative variability was evaluated using standard deviations, interquartile ranges, and confidence intervals rather than Coefficients of Variation, which become mathematically uninformative near zero boundaries.

In microalgae plastic interaction systems, apparent mass changes can also be influenced by surface associated biological materials (Flemming and Wingender, 2010; Harrison et al., 2014; Oberbeckmann et al., 2015). Such processes are particularly relevant for pigmented and additive rich polymers, which provide heterogeneous surface properties that may enhance microbial attachment and retention of organic matrices.

### 3.2 Factorial non-parametric model and interaction analysis

Prior to hypothesis testing, distributional assumptions were evaluated across all experimental groups. Significant departures from normality were identified using the Shapiro–Wilk test (*p* < 0.05). Additionally, Brown–Forsythe Levene’s test revealed significant variance heterogeneity across groups (*F*_(17, 90)_ = 1.86, *p* = 0.032). Because two-way ANOVA assumptions were violated, an Aligned Rank Transform (ART) ANOVA was conducted as a robust non-parametric factorial model to evaluate main and interaction effects (Table 2).

**Table 2:** Summary of aligned rank transform (ART) ANOVA evaluating main and interaction effects of treatment and plastic formulation on gravimetric mass change percentage (WL%).

Term	Df	Df. res	Sum Sq	Sum Sq.res	*F* value	*p*-value	Partial *η*^2^	Effect size interpretation	Significance
Treatment	2	90	26,404.06	52,720.33	22,54	1.16 × 10^−8^	0.334	Large	***
Plastic	5	90	10,518.69	75,704.33	2,50	0.036	0.122	Medium	*
Treatment × Plastic	10	90	19,110.94	45,146.08	3,81	2.60 × 10^−4^	0.297	Large	***

Df, Degrees of freedom; Df.res, Residual degrees of freedom; Sum Sq, Effect sum of squares; Sum Sq.res, Residual sum of squares. Significance codes: ***p < 0.001, *p < 0.05.

The ART ANOVA demonstrated a highly significant main effect for Treatment (*F*_(2, 90)_ = 22.54, *p* < 0.001, $\eta_{p}^{2}$ = 0.334), representing a large effect size. The main effect of Plastic Formulation was also statistically significant (*F*_(5, 90)_ = 2.50, *p* = 0.036, $\eta_{p}^{2}$ = 0.122), corresponding to a medium effect size. Crucially, a highly significant Treatment × Plastic interaction effect was observed (*F*_(10, 90)_ = 3.81, *p* < 0.001, $\eta_{p}^{2}$ = 0.297), accounting for a substantial proportion of the total variance. This strong interaction confirms that microalgal exposure impacts on plastic gravimetric mass loss were non-uniform and strictly dependent on the specific commercial formulation.

Post-hoc pairwise contrasts on aligned ranks were subsequently performed using Tukey’s HSD adjustment to control family wise error rates (Table 3). Pairwise analysis indicated that overall significant differences were driven by specific interactions rather than uniform shifts across all plastic types. Within control groups, significant baseline rank differences were observed between Control Green and Control Black (Estimate = 85.25, t_(90)_ = 4.66, *p* < 0.001) as well as Control Blue and Control Black (Estimate = 77.83, t_(90)_ = 4.26, *p* = 0.0023). Furthermore, significant contrasts were identified between controls and microalgal exposure groups, including Control Gray versus *Chlorella vulgaris* Gray (Estimate = 69.58, t_(90)_ = 3.81, *p* = 0.0116) and Control Green versus *Scenedesmus dimorphus* Black (Estimate = 66.92, t_(90)_ = 3.66, *p* = 0.0189). The vast majority of remaining pairwise contrasts yielded no statistically significant differences (*p* ≥ 0.05), indicating that gravimetric alterations were localized to specific algal polymer combinations.

**Table 3:** Statistically significant *post-hoc* pairwise comparisons on aligned ranks following ART ANOVA (Tukey-adjusted *p* < 0.05).

Contrast/Comparison	Estimate	SE	df	*t*-ratio	*p*-value	Significance
Control Green – Control Black	85.25	18.29	90	4,66	0,0005	***
Control Blue – Control Black	77.83	18.29	90	4,26	0,0023	**
Control Gray – Chlorella Gray	69.58	18.29	90	3,81	0,0116	*
Control Green – Scenedesmus Black	66.92	18.29	90	3,66	0.0189	*
Control Blue – Scenedesmus Black	63.83	18.29	90	3,49	0.0331	*
Control Gray – Chlorella Black	63.42	18.29	90	3,47	0.0357	*

SE, Standard error; df, Degrees of freedom. *Post-hoc* tests were performed using Tukey’s HSD method for family wise error rate control. Significance codes: ***p < 0.001, **p < 0.01, *p < 0.05. Non-significant contrasts (p ≥ 0.05) are omitted for brevity.

### 3.3 Distributional profiles, influence diagnostics, and sensitivity analysis

The distributional patterns and inter replicate dispersion of mass change across experimental groups are illustrated in Figure 1. Most plastic formulations (Blue, Green, Gray, White LDPE, and PetWhite) exhibited tightly clustered observations centered near zero net mass change. Higher dispersion was restricted to specific combinations, notably Black LDPE under *Chlorella vulgaris* and Blue PET under *Scenedesmus dimorphus*. In Black LDPE exposed to *C. vulgaris*, a single biological replicate exhibited an elevated mass loss of 24.14% (Observation ID: 92), whereas remaining replicates in that group clustered near zero or negative values.

**Figure 1:**
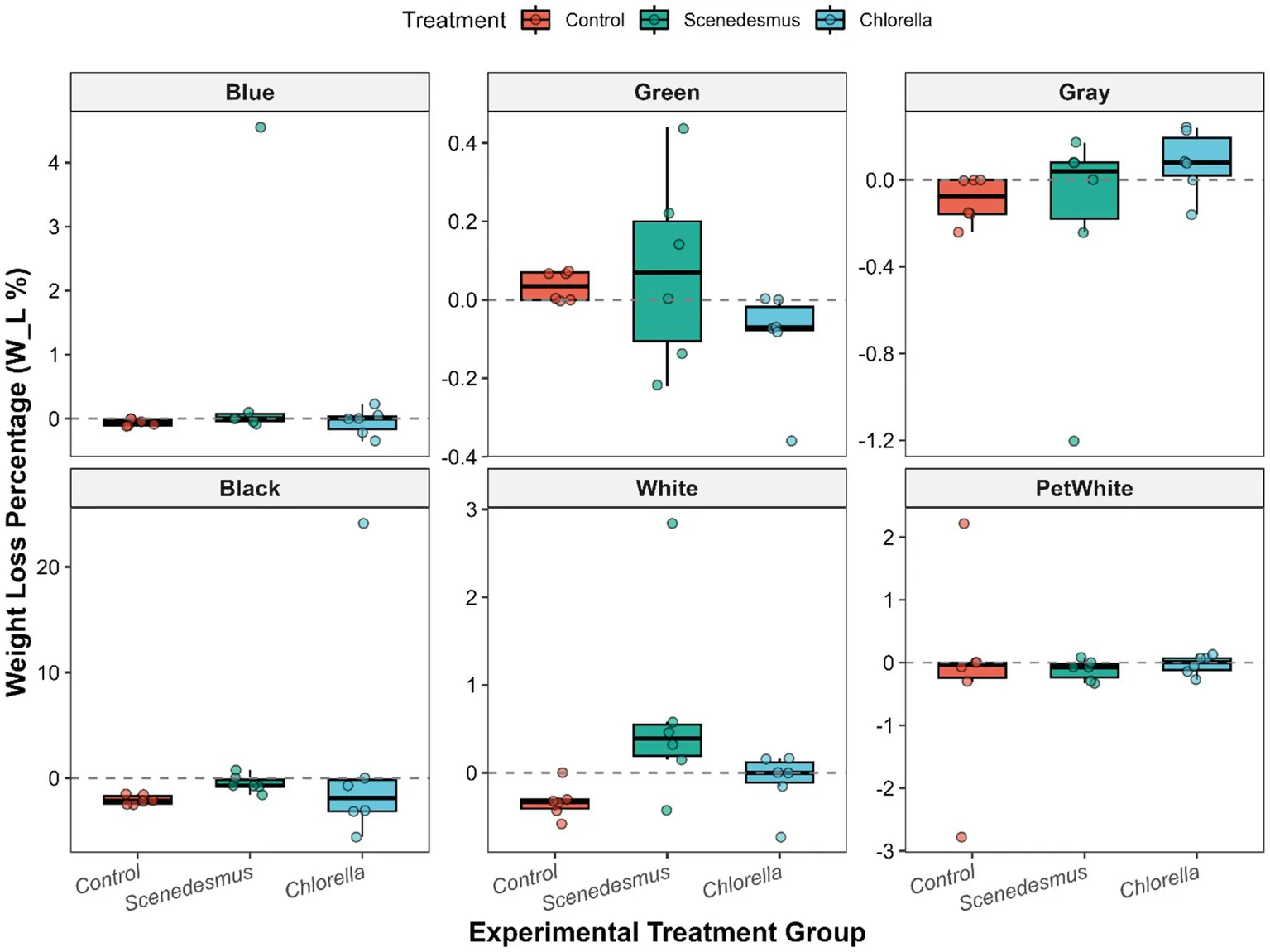
Distribution of weight loss percentage (WL%) across different plastic types and experimental treatments after 100 days of incubation. Boxplots show the median, interquartile range, and range of the data, while individual points represent biological replicates (*n* = 6). The dashed horizontal line indicates zero weight loss. Positive WL% values indicate net mass loss, whereas negative values indicate net mass gain.

The distribution of weight loss varied among plastic types and microalgal treatments. Blue, Green, Gray, White, and PetWhite plastics generally exhibited weight loss values clustered around zero, indicating relatively small net mass changes during the 100 day incubation period. In contrast, Black LDPE mulch film showed the greatest dispersion of observations, reflecting higher variability among biological replicates.

The nonparallel trajectories depicted in the interaction plot (Figure 2) further highlight the formulation dependent response to microalgal exposure. While Control groups remained near zero or slightly negative across materials, *S. dimorphus* promoted positive mass loss in Blue PET and White LDPE, whereas *C. vulgaris* exhibited its primary divergence in Black LDPE.

**Figure 2:**
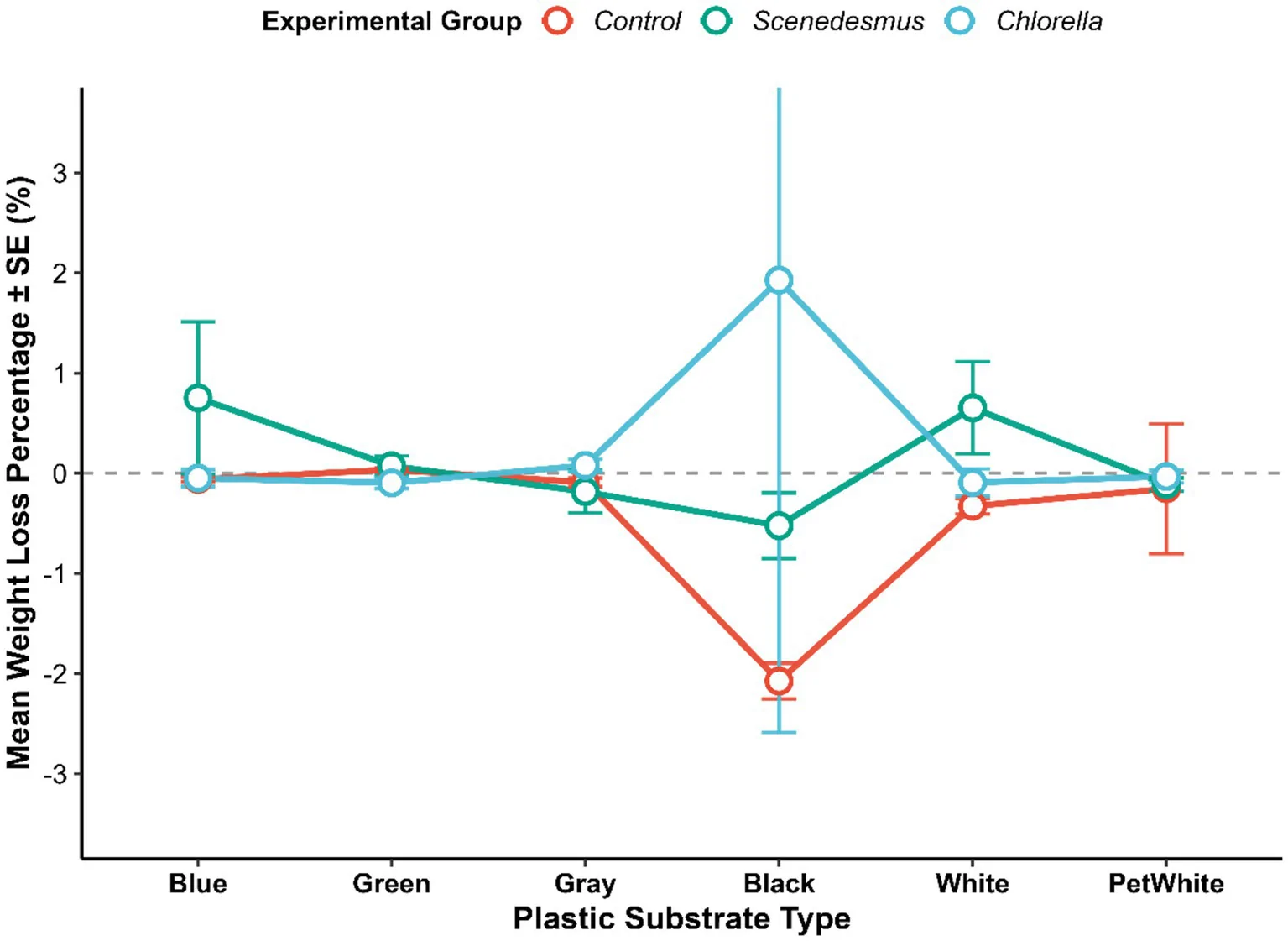
Interaction plot showing the mean weight loss percentage (WL%) ± standard error (SE) of six plastic substrate types following 100 days of incubation under Control, *Scenedesmus dimorphus*, and *Chlorella vulgaris* treatments. The dashed horizontal line represents zero weight loss. Error bars indicate the standard error of six biological replicates (*n* = 6).

To evaluate whether the primary Treatment × Plastic interaction was driven solely by influential observations, a formal sensitivity analysis was performed by rerunning the ART model after excluding the 24.14% extreme value (Table 4). The sensitivity model confirmed that overall scientific conclusions remained robust: the Treatment × Plastic interaction retained high statistical significance (*F* = 4.67, *p* = 2.41 × 10^−5^, $\eta_{p}^{2}$ = 0.344) upon exclusion of the influential replicate. Because diagnostic checks indicated no technical, analytical, or measurement errors, the full dataset (N = 108) was retained for primary reporting.

**Table 4:** Sensitivity analysis: comparison of ART ANOVA interaction model statistics between the full dataset (*N* = 108) and the model excluding the extreme observation (*N* = 107).

Model/Dataset	Included sample size (*N*)	Excluded observation	Interaction *F* value	Interaction *p*-value	Partial η^2^	Conclusion
Full dataset	108	None	3,81	2.60 × 10^−4^	0.297	Significant effect
Sensitivity model	107	ID 92 (Chlorella × Black, 24.14%)	4,67	2.41 × 10^−5^	0.344	Significant effect

Models evaluate the Treatment × Plastic interaction term (df = 10, df_res_ = 90 for full dataset; df = 10, df_res_ = 89 for sensitivity model). Partial η^2^ values quantify effect size.

### 3.4 Fourier transform infrared (FTIR) spectroscopy analysis results

Chemical transformations occurring on the plastic surfaces were investigated using FTIR spectroscopy. Spectra were recorded across 4,000–400 cm^−1^ to evaluate modifications in functional groups relevant to polymer structure and oxidation state. Special attention was focused on the carbonyl, hydroxyl, and aliphatic hydrocarbon regions. FTIR spectroscopy provides a useful analytical approach for identifying changes in surface functional groups associated with polymer oxidation and biological exposure (Wei and Zimmermann, 2017; Mohanan et al., 2020).

#### 3.4.1 Baseline characterization of abiotic control groups

To establish baseline chemical profiles and distinguish biological effects from abiotic background variation, control plastic samples were incubated for 100 days in sterile growth medium in the absence of microalgae and subsequently analyzed by FTIR spectroscopy (Figure 3). The spectra reflected the expected functional group configurations for each polymer class.

**Figure 3:**
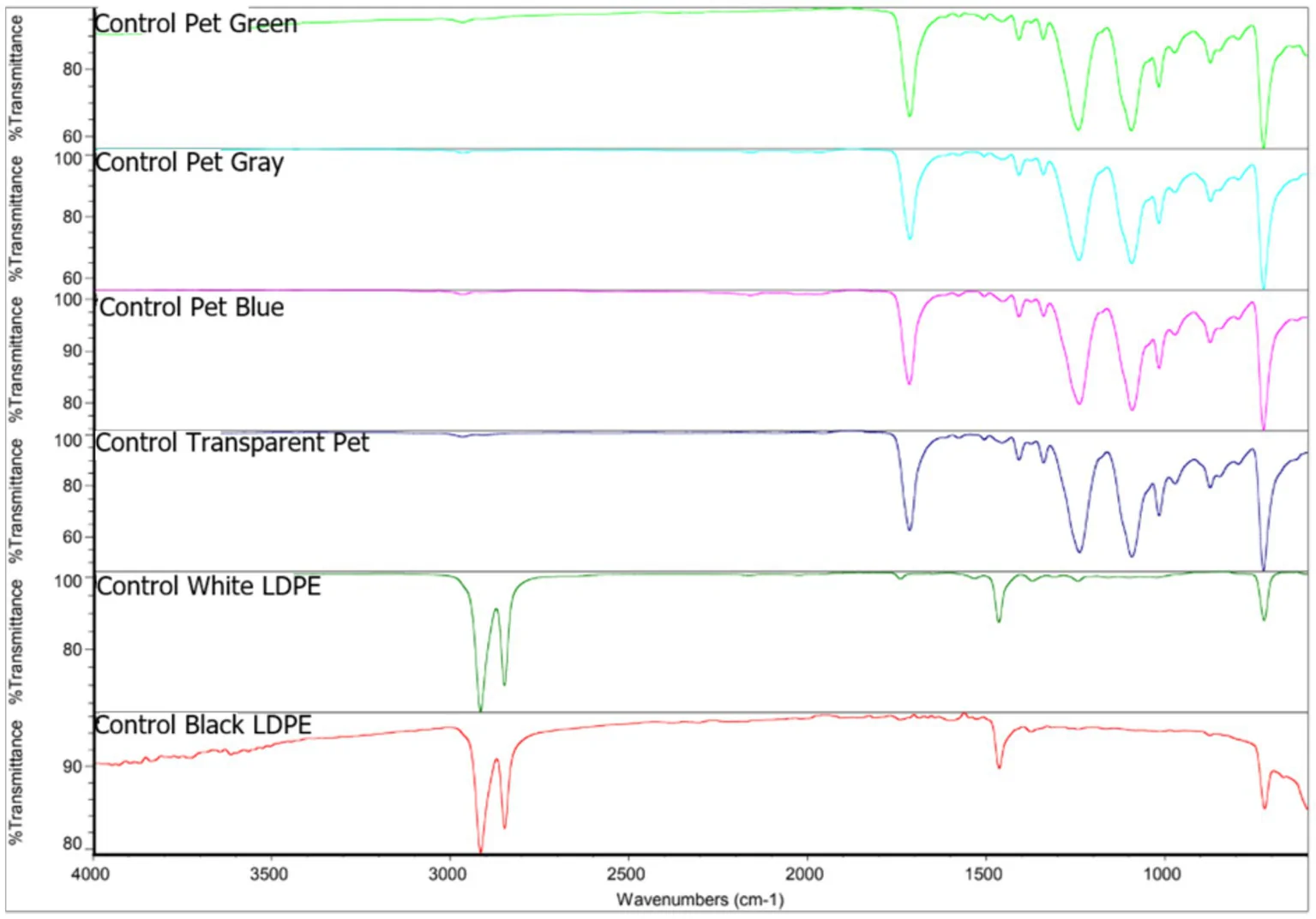
FTIR-ATR spectra of abiotic control plastic formulations after 100 days of incubation in sterile culture medium.

##### 3.4.1.1 Polyester based controls (PET formulations)

The control spectra for unpigmented PET (Control PET) and colored formulations (Control Blue, Control Gray, and Control Green) exhibited virtually identical absorption profiles (Figure 3). All four matrices displayed:

- A strong, sharp absorption band at 1,710–1,715 cm^−1^, corresponding to ester carbonyl (C=O) stretching.
- Prominent peaks at approximately 1,240 cm^−1^ and 1,100 cm^−1^ associated with ester linkage (C-O) stretching vibrations.
- A sharp peak at 725 cm^−1^, representing out of plane aromatic ring C-H bending.

The preservation of these characteristic peaks indicates that no substantial abiotic hydrolysis or other detectable chemical alterations occurred during the 100 day incubation period under sterile conditions.

##### 3.4.1.2 Polyethylene based controls (white and black LDPE films)

Control White (greenhouse film) and Control Black (mulch film) displayed typical aliphatic absorption bands characteristic of Polyethylene (PE) (Figure 3):

- Pronounced aliphatic methylene (CH_2_) doublet stretching vibrations at approximately 2,915 cm^−1^ and 2,845 cm^−1^ corresponding to asymmetric and symmetric C–H stretching modes.
- A characteristic CH_2_ scissoring bending band at 1,460–1,470 cm^−1^ and a long chain methylene rocking band at 715–720 cm^−1^.
- Moderate baseline attenuation in the 1,000–1,200 cm^−1^ region for Control Black, potentially related to additive associated scattering effects.

No newly developed hydroxyl (-OH, ~ 3,400 cm^−1^) or carbonyl (C=O, ~ 1,715 cm^−1^) bands indicative of oxidative modification were detected in the abiotic controls, suggesting that no substantial FTIR detectable oxidative alteration occurred during the 100 day incubation period. These stable control profiles indicate that exposure to the sterile growth medium and incubation conditions did not result in substantial FTIR detectable modification of the polymer structures. Therefore, the abiotic control profiles provide a baseline against which FTIR detectable surface chemical changes in the *Scenedesmus dimorphus* and *Chlorella vulgaris* inoculated treatments can be evaluated, allowing differences observed following microalgal exposure to be distinguished from changes associated with the growth medium and incubation conditions alone.

#### 3.4.2 FTIR analysis of *Scenedesmus dimorphus* exposed plastic samples

After 100 days of incubation with *Scenedesmus dimorphus*, the investigated plastic formulations retained the major spectral features characteristic of their respective polymer matrices, although some differences in peak intensity and baseline characteristics were observed (Figure 4). These observations are interpreted as indications of localized surface level physicochemical changes rather than evidence of polymer degradation, depolymerization, or mineralization.

**Figure 4:**
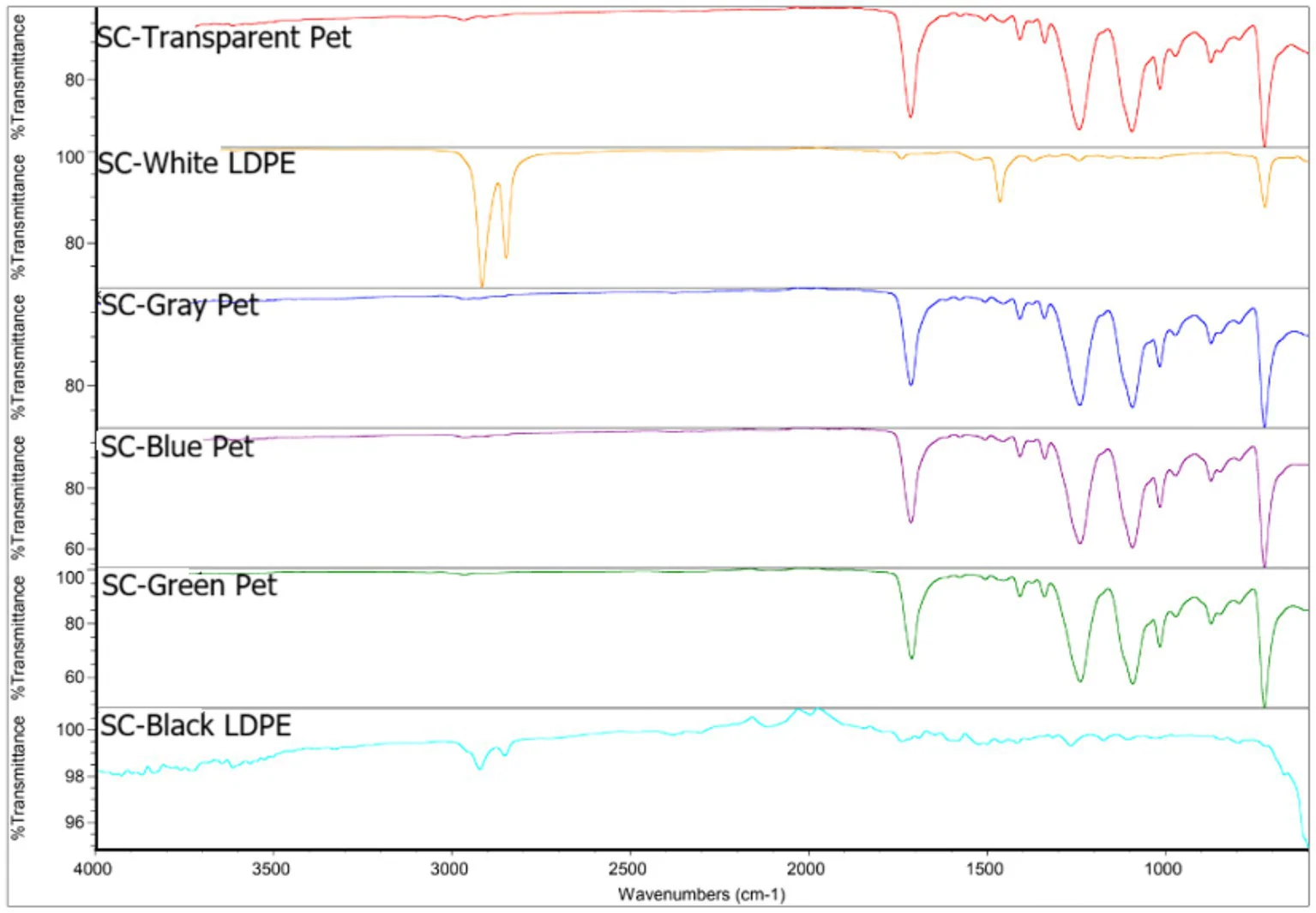
FTIR-ATR spectra of the investigated plastic formulations after 100 days of exposure to *Scenedesmus dimorphus*.

##### 3.4.2.1 Polyester based formulations

In the carbonyl region (approximately 1,710–1,715 cm^−1^), all PET formulations exhibited a distinct absorption band attributable to ester carbonyl (C=O) stretching. The position of this band remained comparable among the PET formulations after exposure. Minor differences in relative peak intensity and baseline characteristics were observed, particularly for the blue and green formulations, relative to their corresponding abiotic controls (Figure 4).

In the fingerprint region, the characteristic ester related C–O stretching bands around 1,240 and 1,100 cm^−1^, together with the aromatic C–H out of plane bending feature near 725 cm^−1^, remained detectable in all PET samples. Variations in spectral intensity within the approximately 1,240–1,000 cm^−1^ region may indicate differences in surface composition, surface coverage, or local chemical environment following incubation. However, because no substantial displacement or disappearance of the principal PET associated bands was observed, the FTIR spectra do not provide sufficient evidence to conclude that extensive alteration of the PET polymer structure occurred.

##### 3.4.2.2 Polyethylene based formulations

The white LDPE formulation (SC-White LDPE) exhibited the characteristic aliphatic methylene (CH₂) stretching bands at approximately 2,915 and 2,845 cm^−1^, together with the CH₂ scissoring band near 1,465 cm^−1^ and the rocking band near 718 cm^−1^ (Figure 4). No clearly distinguishable new absorption bands attributable to hydroxyl (–OH) or carbonyl (C=O) groups were observed in the spectra. Thus, within the sensitivity and resolution of the FTIR-ATR analysis, no pronounced change in the principal spectral features of the white LDPE formulation was detected after exposure.

The black LDPE formulation retained the characteristic aliphatic C–H stretching bands near 2,915 and 2,845 cm^−1^, although these features were accompanied by increased baseline variation and broader spectral irregularities across parts of the midinfrared region (approximately 3,500–1,500 cm^−1^) (Figure 4). Such spectral characteristics may be associated with the optical and surface related properties of the commercial black formulation and/or with material deposited on the polymer surface, including components of the associated biofilm. Because the chemical composition and concentration of the additives or colorants in the commercial formulations were not independently determined, these spectral features cannot be assigned specifically to carbon black or to a particular additive.

Exposure to *S. dimorphus* was associated with relatively subtle changes in FTIR spectral intensity and baseline characteristics, while the principal polymer associated absorption bands remained detectable in the investigated PET and LDPE formulations. The FTIR results therefore support the occurrence of localized surface level physicochemical changes following algal exposure but do not, by themselves, demonstrate polymer chain scission, depolymerization, or complete biodegradation. These findings should be considered together with the gravimetric and SEM observations when evaluating the extent of plastic surface alteration.

#### 3.4.3 FTIR analysis of *Chlorella vulgaris* exposed samples

Following 100 days of incubation with *Chlorella vulgaris*, the FTIR-ATR spectra of the commercial plastic formulations were examined to identify potential changes in their characteristic surface associated functional groups (Figure 5). Overall, the principal absorption bands characteristic of the respective polymer matrices were retained after microalgal exposure. The observed differences were mainly associated with variations in band intensity and baseline features rather than the appearance of pronounced new absorption bands.

**Figure 5:**
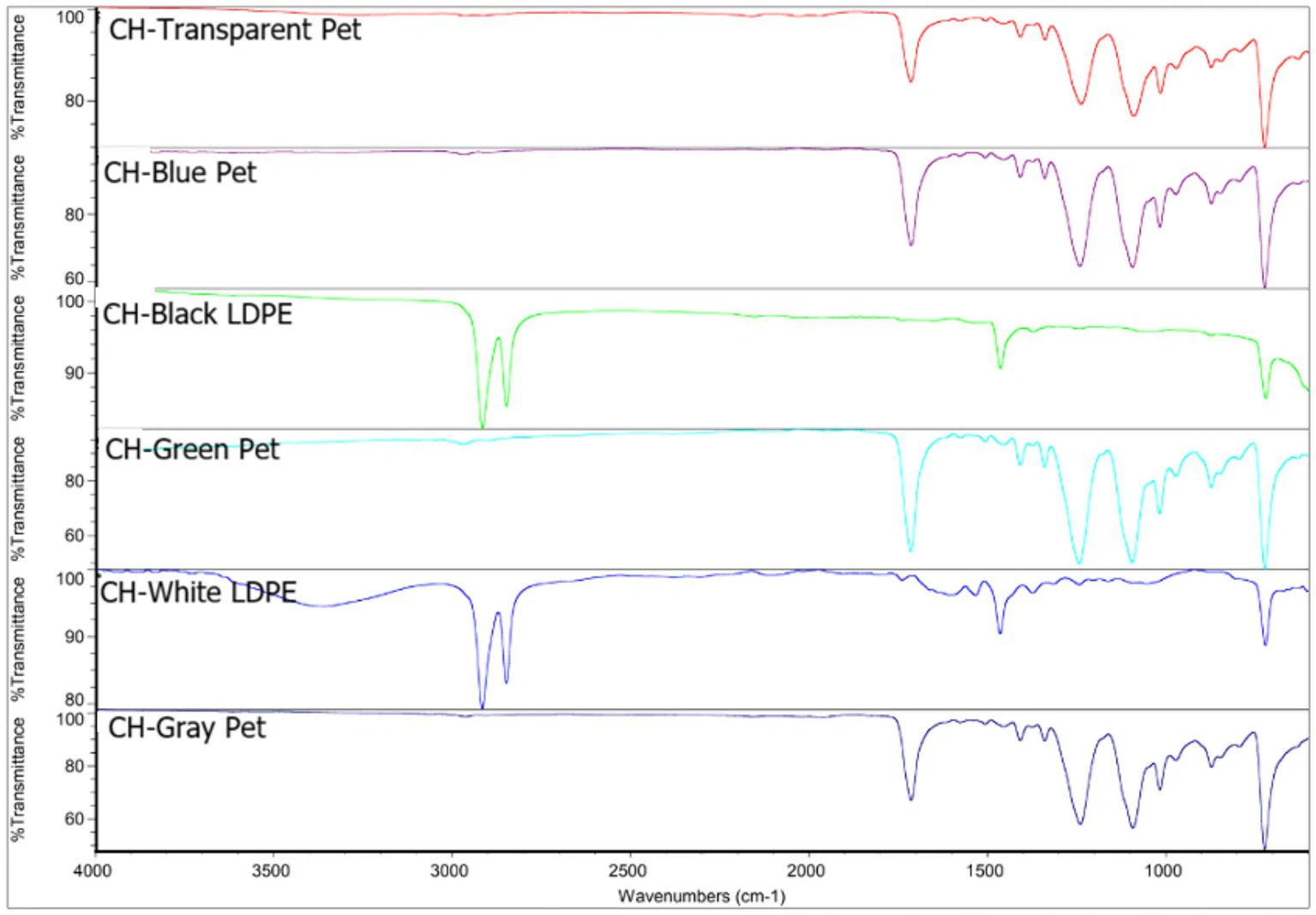
FTIR-ATR spectra of plastic formulations after 100 days of exposure to *Chlorella vulgaris*.

##### 3.4.3.1 Polyester-based formulations (PET: blue, green, and gray)

###### 3.4.3.1.1 Carbonyl region (1,710–1,715 cm^−1^)

All PET formulations retained the characteristic ester carbonyl (C=O) stretching band in the region of approximately 1,710–1,715 cm^−1^. Differences in band intensity and baseline profile were observed among the pigmented PET formulations relative to the corresponding abiotic controls. However, the position and overall presence of the characteristic carbonyl band remained evident, indicating that the principal ester related functional group was retained following the 100 day exposure period.

###### 3.4.3.1.2 Fingerprint region (1,400–900 cm^−1^)

The characteristic PET fingerprint features were also retained, including the C–O stretching bands near 1,240 and 1,100 cm^−1^. The aromatic C–H out of plane bending band around 725 cm^−1^ remained detectable in the PET formulations. Some differences in relative band intensity were more apparent in the blue and green samples; however, these changes were not accompanied by pronounced alterations in the main spectral pattern. Thus, the FTIR spectra did not indicate substantial changes in the characteristic functional group profile of the PET matrices under the applied experimental conditions.

##### 3.4.3.2 Polyethylene based formulations (white and black LDPE)

###### 3.4.3.2.1 Hydroxyl region (~3,300–3,400 cm^−1^)

The white LDPE greenhouse film (CH-White LDPE) exhibited a broad, low intensity absorption feature in the approximately 3,300–3,400 cm^−1^ region (Figure 5). This region is commonly associated with O–H stretching vibrations. The observed feature may reflect the presence of hydroxyl containing species associated with the plastic surface and/or residual moisture or organic material following microalgal exposure. Therefore, the band is considered indicative of a change in the surface associated spectral profile, although its specific origin cannot be assigned solely on the basis of FTIR-ATR measurements.

###### 3.4.3.2.2 Aliphatic region (2,915–2,845 cm^−1^)

Both black and white LDPE formulations retained the characteristic aliphatic hydrocarbon profile, including the asymmetric and symmetric CH₂ stretching bands near 2,915 and 2,845 cm^−1^, respectively. The CH₂ bending vibration around 1,465 cm^−1^ and the rocking vibration near 718 cm^−1^ were also retained. The black LDPE sample showed a relatively stable baseline profile, with no pronounced additional absorption bands detected within the measured spectral range.

Exposure to *C. vulgaris* for 100 days was associated with limited changes in the FTIR-ATR spectral profiles of the investigated plastic formulations. The major characteristic bands of both PET and LDPE were retained, while localized differences in band intensity and baseline features were observed. The broad O–H-associated feature detected for white LDPE may indicate the presence of hydroxyl containing or surface associated organic species after microalgal exposure; however, FTIR-ATR alone does not allow the specific origin of this feature to be established. These findings therefore suggest that the principal functional group signatures of the investigated polymers remained detectable following exposure, while some surface associated spectral modifications may have occurred.

#### 3.4.4 Core systemic insights: *Scenedesmus dimorphus* vs. *Chlorella vulgaris*

Comparison of the two independent microalgal systems revealed distinct differences in the extent and trajectory of early stage polymer surface modification (Table 5).

**Table 5:** Comparative early stage surface modification characteristics of *Scenedesmus dimorphus* and *Chlorella vulgaris*.

Spectral feature	*Scenedesmus dimorphus* system (Figure 4)	*Chlorella vulgaris* system (Figure 5)
Carbonyl (C=O) region	Moderate band broadening, consistent with changes in oxygen containing functional groups.	More pronounced peak intensification and band broadening around ~1,710 cm^−1^, suggesting greater changes in the carbonyl associated region.
Aliphatic (C–H) region	Moderate attenuation of bands within the 2,850–2,950 cm^−1^ region.	More pronounced decrease in band intensity within the 2,850–2,950 cm^−1^ region, suggesting greater alteration of aliphatic C–H associated signals.
Fingerprint region (1,400–900 cm^−1^)	Changes varied with polymer pigmentation, with more evident alterations in green and blue samples and limited changes in black samples.	Spectral changes were observed across a broader range of samples, although baseline variability was also evident.
Overall spectral response	Changes were consistent with early stage surface chemical modification during prolonged exposure.	The stronger spectral response was consistent with a comparatively greater degree of early stage surface chemical modification under the tested conditions.

Both microalgal systems produced measurable changes in the FTIR spectra of the investigated polymer surfaces, indicating that prolonged microalgal exposure was associated with progressive chemical modification of the polymer interfaces. However, the *Chlorella vulgaris* system exhibited more pronounced changes in carbonyl associated and aliphatic spectral regions, suggesting a greater extent of early stage surface modification under the tested conditions.

These observations should not be interpreted as evidence that either microalgal system completely overcomes the protective characteristics of commercial plastics. Rather, the results support a progressive deterioration model in which microalgae polymer interactions may contribute to surface conditioning, functional group modification, and physicochemical weakening of the polymer interface. In this context, surface associated processes, including biofilm formation and microalgal attachment, may act as important precursor mechanisms that increase the susceptibility of PET and PE surfaces to subsequent degradation processes during prolonged environmental exposure. The stronger spectral response observed for *C. vulgaris* further suggests that its interaction with the polymer surface may promote a comparatively greater degree of early stage chemical modification under the experimental conditions examined.

### 3.5 Scanning electron microscopy (SEM) analysis results

Surface morphological changes following microalgal exposure were examined using a Zeiss EVO 15 scanning electron microscope equipped with a lanthanum hexaboride (LaB6) electron source. Following incubation, plastic fragments were dehydrated through a graded ethanol series before being sputter coated with a thin conductive metal layer to reduce surface charging during imaging. SEM observations were conducted at an accelerating voltage of 15.00 kV, with the working distance (WD) maintained between 8.3 and 9.5 mm. Micrographs were evaluated for differences in surface texture and for localized morphological features, including irregularities, cracks, pits, cavities, and delamination like structures. The SEM analysis was used to characterize surface morphology and compare microalgae exposed samples with their corresponding abiotic controls. Because sample preparation procedures and drying, may influence the appearance of thin polymer surfaces, the observed morphological features were interpreted as surface alterations rather than as direct evidence of polymer biodegradation, depolymerization, or mineralization.

SEM analysis was performed after the 100 day incubation period to evaluate differences in the surface morphology of the plastic samples. Compared with the corresponding abiotic controls, the microalgae exposed plastics exhibited varying degrees of surface morphological alteration depending on the plastic formulation and microalgal species. The observed differences included changes in surface texture and localized irregularities, with some samples additionally exhibiting crack like, pit like, cavity like, or delamination like features. These observations indicate that the extent and nature of surface alteration varied among the tested plastic formulations following microalgal exposure. However, the presence of these features was not interpreted as definitive evidence of polymer degradation because SEM observations alone cannot establish polymer chain breakdown or mineralization.

The abiotic control PET samples generally exhibited relatively intact surface morphologies after the 100 day incubation period (Figure 6). Control-PET, Control-Blue, and Control-Green showed predominantly smooth surfaces with limited localized irregularities. In contrast, Control-Gray exhibited a comparatively more granular and heterogeneous surface texture. This difference was considered a baseline morphological characteristic of the tested commercial formulation rather than being attributed to a specific pigment or additive, as the chemical composition and concentration of the formulation components were not determined in the present study. Small particulate or irregular features were visible on some control surfaces; however, no pronounced cracks, pits, delamination, or extensive surface discontinuities were apparent under the examined SEM conditions.

**Figure 6:**
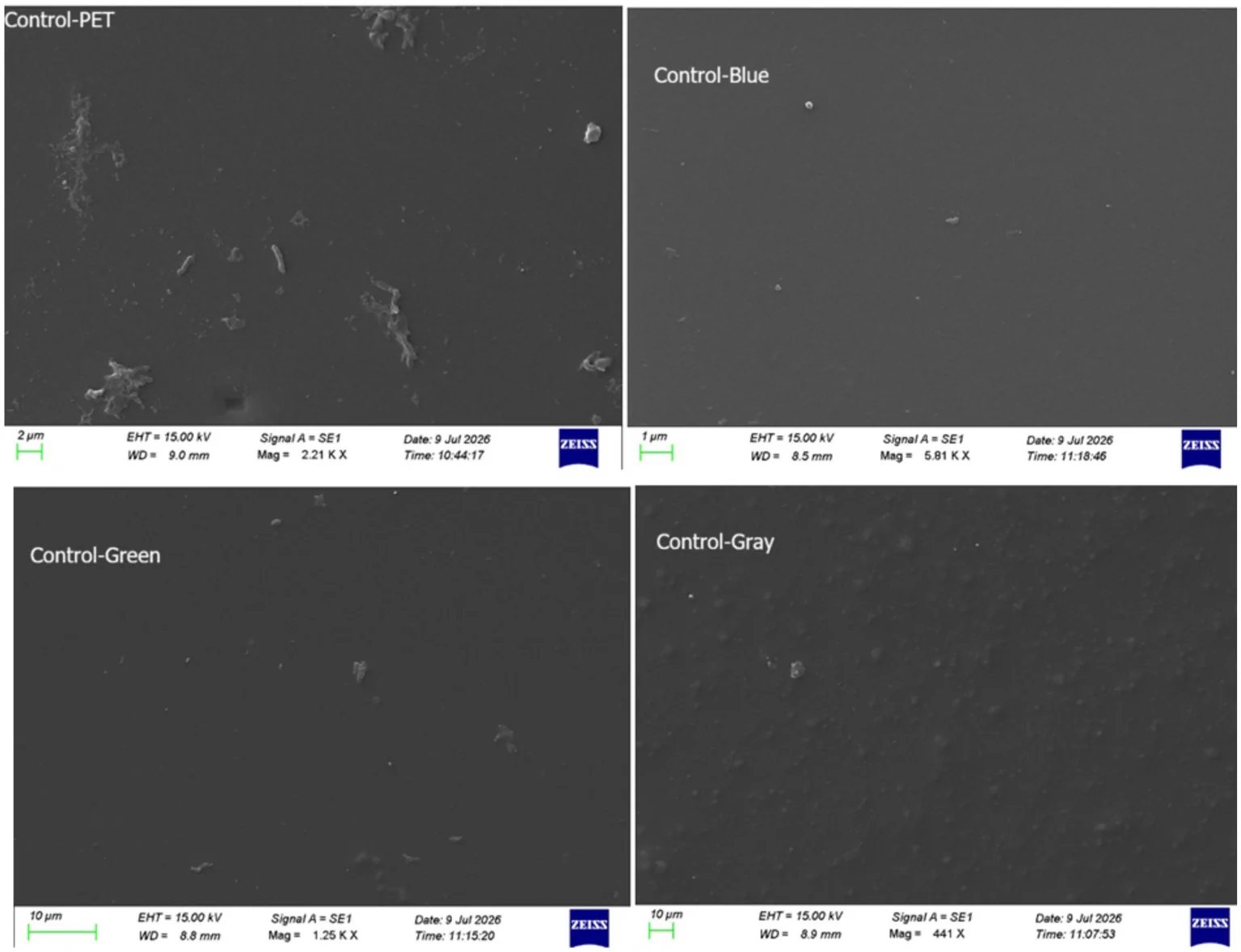
SEM micrographs of the abiotic control PET formulations after 100 days of incubation. Control-PET, transparent PET; Control-Blue, blue PET; Control-Green, green PET; Control-Gray, gray PET. The control samples exhibited predominantly intact surface morphologies, with limited localized irregularities and no pronounced cracks, pits, or delamination under the examined SEM conditions.

The agricultural LDPE control samples also exhibited distinct baseline surface morphologies (Figure 7). The Control Greenhouse White sample showed a predominantly continuous and relatively smooth surface with several small, rounded pore like features distributed across the examined area. The Control-Black sample exhibited a comparatively rougher and more heterogeneous surface texture characterized by fine irregular and elongated features. These differences were observed under abiotic conditions and therefore provide important formulation specific morphological references for comparison with the corresponding microalgae exposed samples. No pronounced cracks, extensive cavities, or delamination were apparent in either control sample under the examined SEM conditions.

**Figure 7:**
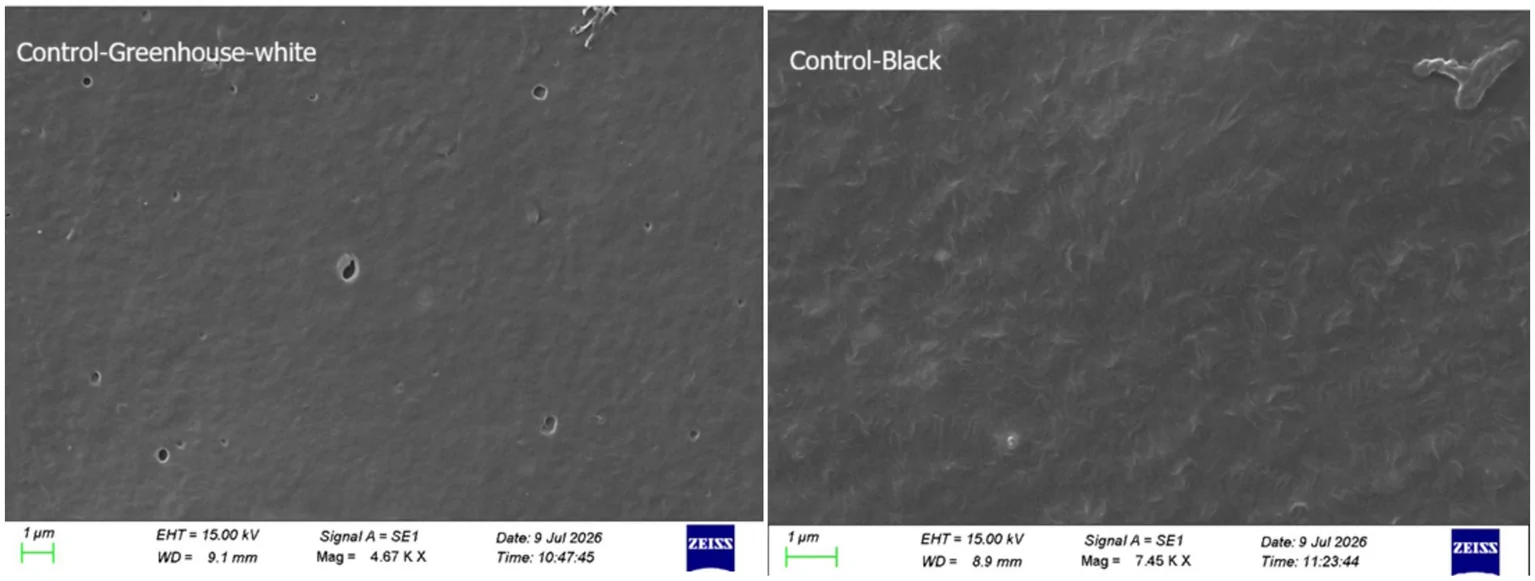
SEM micrographs of the abiotic control agricultural LDPE samples after 100 days of incubation. Control-Greenhouse White, greenhouse covering film; Control-Black, black mulch film. The control samples exhibited distinct baseline surface morphologies, with a relatively smooth surface and localized pore like features in the greenhouse white LDPE and a comparatively rougher, heterogeneous texture in the black LDPE.

SEM analysis revealed distinct surface morphological features in all PET formulations following incubation with *Scenedesmus dimorphus* (Figure 8). The transparent PET sample (SC-PET) exhibited localized peeling and shallow surface cavities, together with linear grooves and minor surface discontinuities. In contrast, the blue PET formulation (SC-Blue) showed more extensive surface disruption, characterized by pronounced roughening, irregular cavities, fissures, and interconnected fractured regions. These features indicate a comparatively greater degree of visible surface alteration in SC-Blue among the formulations examined. The green PET sample (SC-Green) displayed localized delamination and tearing, accompanied by irregular surface discontinuities. The gray PET formulation (SC-Gray) exhibited irregular crater like depressions and porous, fractured surface features, indicating localized disruption of the surface structure.

**Figure 8:**
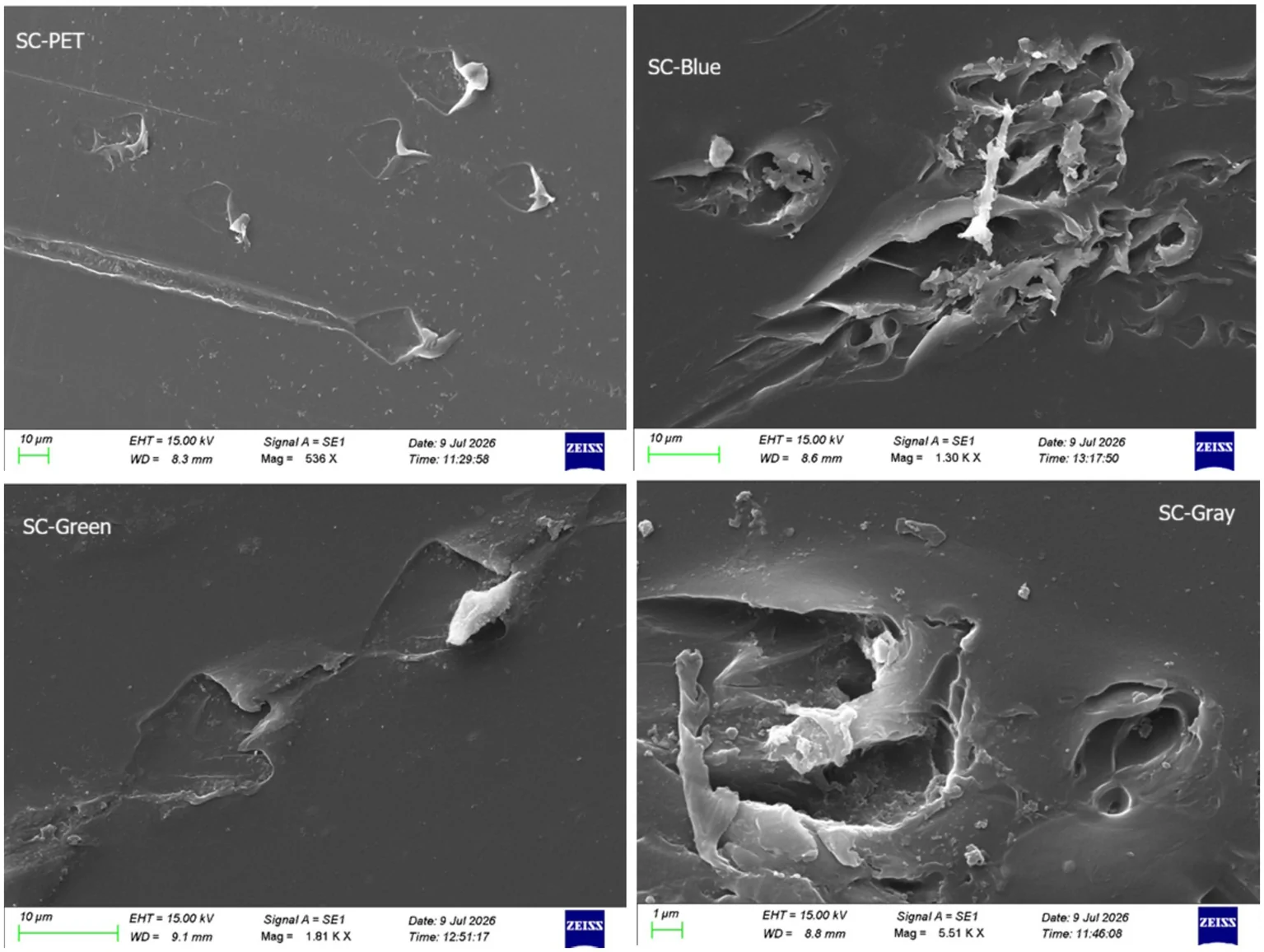
Scanning electron microscopy (SEM) images showing the surface morphology of PET formulations following incubation with *Scenedesmus dimorphus*. SC-PET, transparent PET; SC-Blue, blue PET; SC-Green, green PET; and SC-Gray, gray PET. Distinct morphological features, including surface roughening, cavities, fissures, localized delamination, tearing, and porous or crater-like structures, were observed among the PET formulations.

The SEM observations demonstrate formulation dependent differences in the surface morphology of PET following incubation with *S. dimorphus*. However, because the micrographs were obtained at different magnifications and the present observations are qualitative, the apparent differences in surface alteration should be interpreted as morphological evidence rather than as a quantitative measure of degradation. The observed surface features may reflect physicochemical interactions and/or biological activity associated with incubation with *S. dimorphus*; therefore, SEM morphology alone does not establish polymer chain degradation or mineralization.

The agricultural LDPE films exhibited distinct surface morphological features following incubation with *Scenedesmus dimorphus* (Figure 9). The greenhouse white film showed longitudinal cracks, localized peeling, and areas of surface flaking, indicating localized disruption of the film surface. The black mulch film exhibited a prominent localized cavity accompanied by irregular surface erosion and deformation in the surrounding region. Overall, both agricultural LDPE formulations displayed visually apparent surface alterations following incubation with *S. dimorphus*, although the type and extent of the observed morphological features differed between the two film formulations. These observations provide qualitative evidence of surface modification.

**Figure 9:**
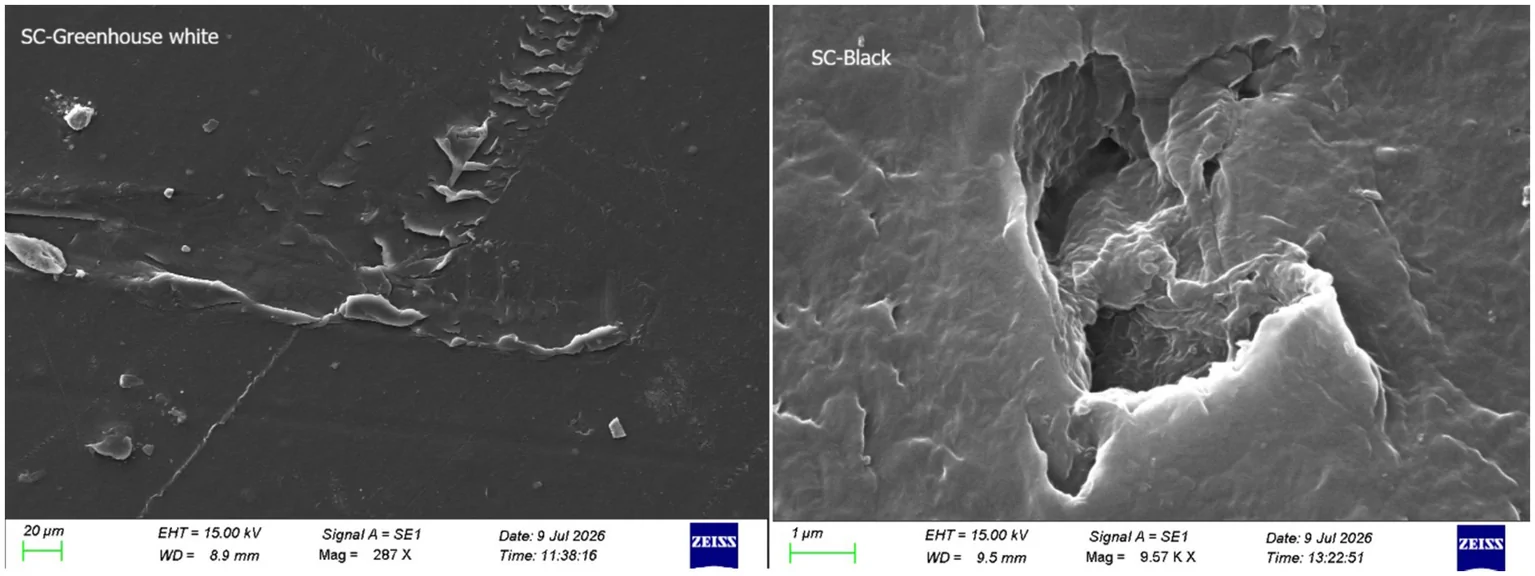
SEM micrographs showing surface morphological changes in agricultural LDPE films following incubation with *Scenedesmus dimorphus*. SC-Greenhouse white exhibited surface cracking and localized flaking, whereas SC-Black showed a localized cavity associated with irregular surface erosion.

Comparable but distinct surface alterations were observed after incubation with *Chlorella vulgaris* (Figure 10). The transparent PET sample exhibited elongated grooves, localized peeling, and shallow cavities. The blue PET formulation showed a localized depression associated with partial detachment of the outer surface layer. The green PET sample displayed angular cracks, localized flaking, and delamination, whereas the gray PET formulation contained a large crater like depression with an irregular internal morphology.

**Figure 10:**
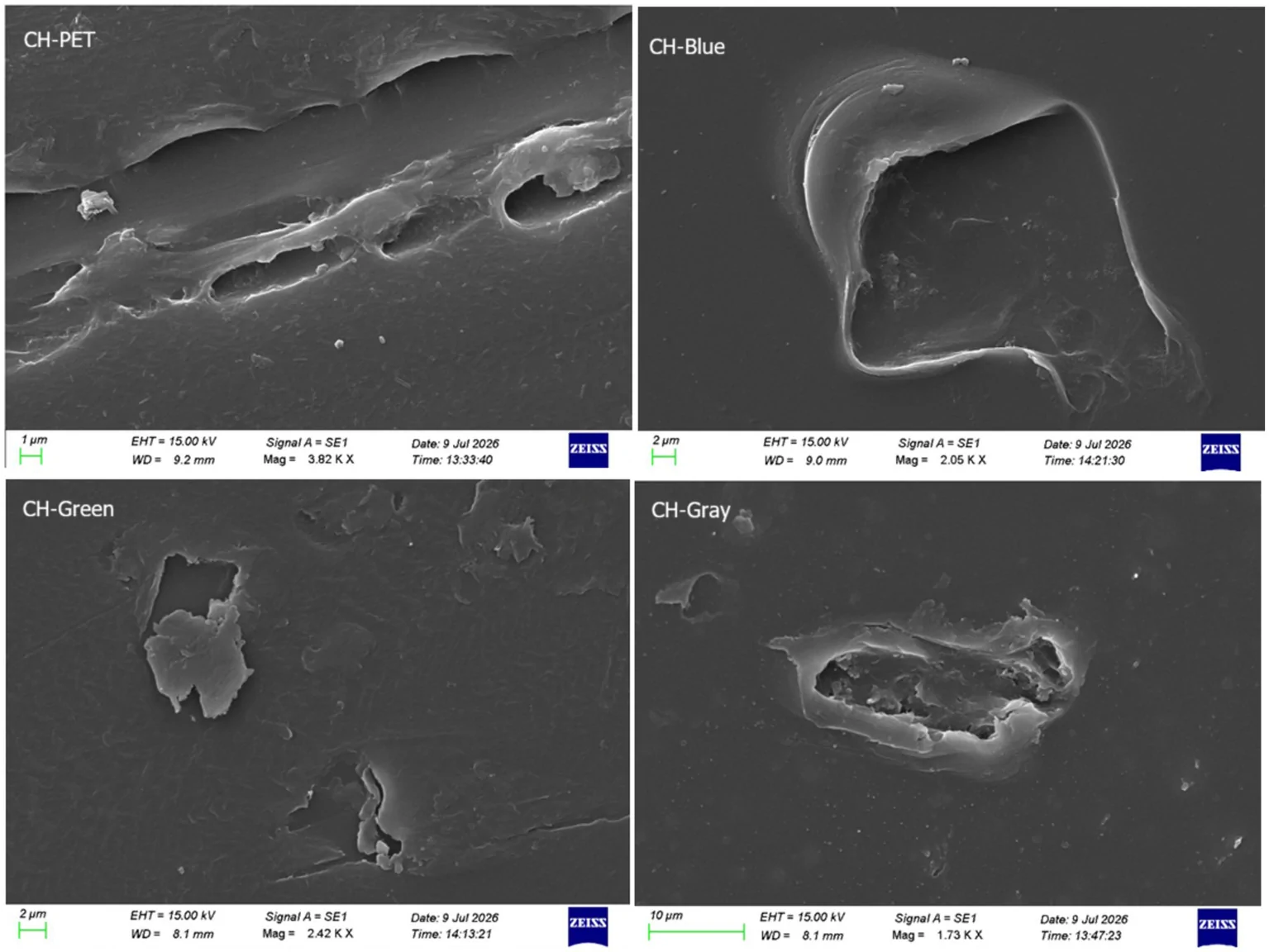
SEM micrographs showing surface morphological changes in PET formulations following incubation with *Chlorella vulgaris*. Localized peeling, cavities, cracks, and delamination were observed, with the extent of alteration differing among the PET formulations.

The agricultural LDPE films exposed to *C. vulgaris* also exhibited distinct surface morphological alterations (Figure 11). The greenhouse white film showed multiple surface cracks, localized delamination, and flaking across the polymer surface. In contrast, the black mulch film exhibited a deep localized cavity with irregular internal erosion features, accompanied by surface folds and fine grooves surrounding the affected area. Overall, SEM observations revealed distinct surface morphological changes in both PET and LDPE formulations following incubation with the two microalgal species, although the extent and pattern of these alterations varied among the plastic formulations.

**Figure 11:**
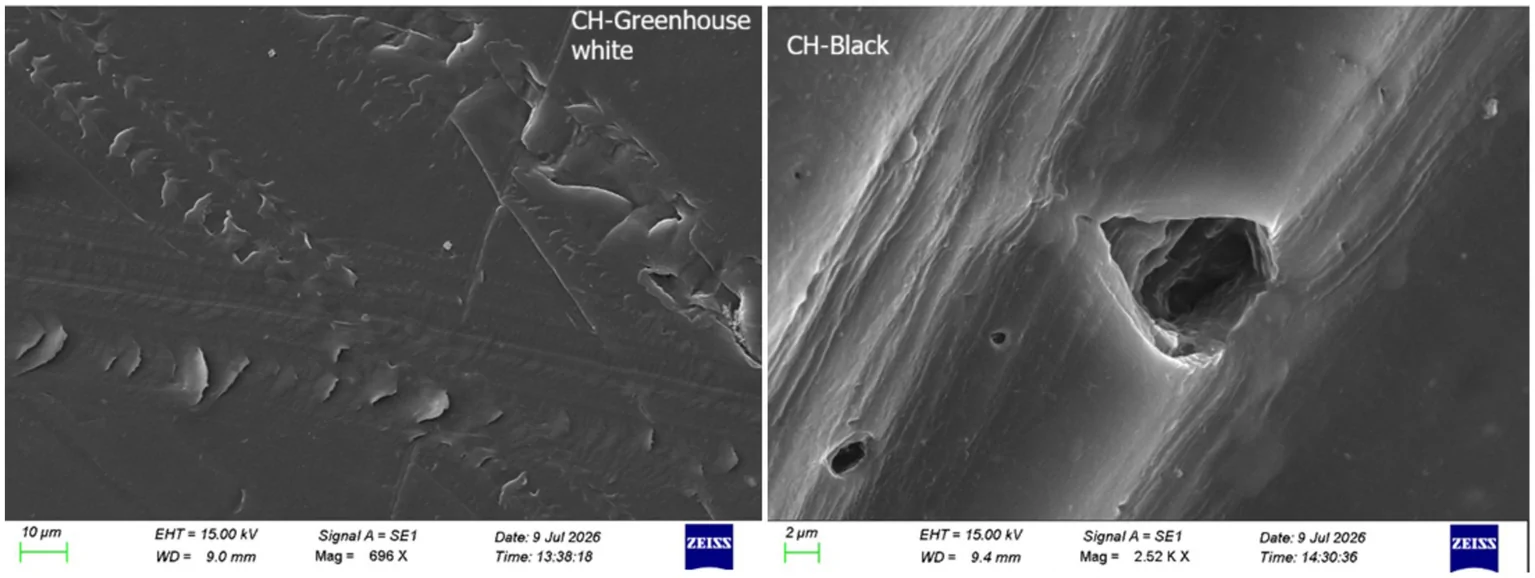
SEM micrographs showing surface morphological changes in agricultural LDPE films following incubation with *Chlorella vulgaris*. The greenhouse white film exhibited surface cracking and delamination, whereas the black mulch film showed a localized cavity with surrounding surface grooves and irregular erosion features.

## 4 Discussion

### 4.1 Microalgal colonization and early stage plastic surface modification

The present study demonstrated that both *Scenedesmus dimorphus* and *Chlorella vulgaris* interacted with commercial PET and LDPE plastics during the 100 day incubation period, resulting in measurable physicochemical and morphological changes on the polymer surfaces. Although the observed gravimetric changes were generally modest, the combined evidence obtained from gravimetric measurements, FTIR spectroscopy, and surface morphology supports the occurrence of surface associated physicochemical modification following microalgal exposure rather than extensive polymer degradation or mineralization. Conventional petroleum based plastics possess highly stable carbon or aromatic ester structures that limit rapid microbial transformation; therefore, early changes at plastic surfaces are commonly associated with surface conditioning, microbial attachment, oxidation, and localized structural alteration rather than complete polymer breakdown (Shah et al., 2008; Wei and Zimmermann, 2017; Mohanan et al., 2020).

The relatively small changes observed in weight loss should therefore be interpreted as net mass changes rather than as a direct measure of biological degradation. Previous studies have emphasized that initial microbial interactions with plastics may occur predominantly at the polymer surface before substantial changes in total polymer mass become detectable (Urbanek et al., 2018; Roy and Chakraborty, 2024). In the present study, FTIR spectra showed changes in functional groups associated with oxygen containing surface chemistry in several plastic types. These observations are consistent with previous reports indicating that microalgal interactions can contribute to physicochemical conditioning of plastic surfaces and may influence subsequent interactions with other microorganisms (Hadiyanto et al., 2022). However, the FTIR results do not by themselves establish polymer chain scission, depolymerization, or mineralization.

Microalgae are increasingly recognized as important components of plastisphere communities. Their production of extracellular polymeric substances (EPS), photosynthetic modification of local oxygen concentrations, and potential effects on local pH may influence the physicochemical conditions at plastic surfaces and affect microbial attachment (Brasil et al., 2017; Junior et al., 2020). In this context, microalgae may contribute to surface conditioning and to the development of microenvironments that support subsequent microbial interactions rather than functioning as complete degraders of conventional synthetic polymers. The present findings therefore support a role for microalgal exposure in early stage surface modification of plastic materials under the experimental conditions examined.

SEM observations revealed localized changes in surface morphology, including roughening, cracks, cavities, and delamination like features in several PET and LDPE samples exposed to the microalgae. These features were generally less pronounced or absent in the corresponding abiotic controls, indicating differences in surface morphology following microalgal exposure. However, such morphological features cannot be considered independent evidence of biological erosion or polymer degradation.

### 4.2 Influence of commercial plastic formulations on early stage surface modification

One of the important findings of this study was that the magnitude of surface modification varied among the tested commercial plastic formulations. The colored PET samples, particularly the blue and green formulations, exhibited more pronounced FTIR alterations than transparent PET, whereas the black agricultural LDPE showed comparatively limited physicochemical modification. These observations indicate that the response of plastics to microalgal exposure varied among commercial formulations. However, because the chemical identities and concentrations of the pigments and additives were not determined in the present study, the observed differences cannot be attributed to specific pigments or additives.

Commercial plastics may contain various formulation components, including pigments, stabilizers, antioxidants, fillers, plasticizers, and ultraviolet protective compounds, which can influence the physicochemical properties and environmental behavior of polymer materials (Dilara and Briassoulis, 2000; Wei and Zimmermann, 2017). Such components may affect surface characteristics, weathering behavior, microbial attachment, and susceptibility to physicochemical modification. Consequently, differences observed among the tested materials should be considered formulation dependent responses rather than direct evidence of a specific additive mediated mechanism.

The greater spectral modifications observed in the blue and green PET formulations may reflect differences in the physicochemical characteristics of these commercial materials. Surface properties such as hydrophobicity, roughness, light absorption, and additive composition can influence microbial attachment and surface reactions. Nevertheless, because the present study did not chemically characterize the commercial formulations, these potential mechanisms remain hypotheses rather than experimentally demonstrated explanations. The results therefore support the importance of considering commercial formulation as a factor when evaluating microalgal interactions with plastic materials.

The black LDPE mulch film exhibited relatively limited FTIR modification throughout the incubation period. Agricultural black polyethylene films commonly contain carbon black, which can function as a pigment and ultraviolet stabilizing component. Carbon black has been reported to absorb ultraviolet radiation and contribute to protection against photooxidative processes, thereby potentially reducing the formation of oxygen containing functional groups (Dilara and Briassoulis, 2000; Yao et al., 2022). The comparatively stable FTIR spectra observed for the black LDPE in the present study are consistent with the protective behavior previously associated with carbon black containing polyethylene formulations. However, because the composition of the commercial mulch film was not independently determined, this interpretation should be regarded as a possible formulation related explanation rather than direct evidence of a carbon black specific mechanism.

These findings emphasize that commercial formulation may contribute substantially to differences in plastic surface responses under microalgal exposure. Studies that classify plastics solely according to polymer type may therefore overlook variation arising from formulation characteristics. Further studies incorporating chemical characterization of pigments and additives would be required to determine the specific contribution of individual formulation components.

### 4.3 Comparison between PET and agricultural LDPE plastics

Distinct surface modification patterns were observed between PET packaging materials and agricultural LDPE films. PET generally exhibited relatively limited changes in comparison with some of the agricultural LDPE formulations, whereas LDPE showed greater variability among commercial materials. These differences may reflect the distinct physicochemical characteristics and formulation properties of the tested polymers.

PET contains aromatic ester linkages and generally exhibits high crystallinity, mechanical strength, and chemical resistance. These characteristics can limit microbial accessibility and contribute to its persistence under environmental conditions (Tokiwa et al., 2009; Wei and Zimmermann, 2017). Consistent with these characteristics, the transparent PET samples showed relatively limited alterations in the ester and carbonyl regions of the FTIR spectra, suggesting that detectable surface associated chemical modification remained limited after 100 days of incubation.

In contrast, polyethylene consists predominantly of long aliphatic carbon chains and lacks readily hydrolysable functional groups. Although this structure also contributes to resistance to microbial transformation, oxidative modification of the polymer surface can introduce oxygen containing functional groups and alter surface properties, potentially affecting subsequent microbial attachment (Shah et al., 2008; Urbanek et al., 2018). The variability observed among the agricultural LDPE formulations in the present study may therefore reflect differences in commercial formulation and surface characteristics in addition to the intrinsic properties of polyethylene.

The contrasting responses of PET and LDPE observed in this study are consistent with previous reports indicating that polymer structure, crystallinity, surface characteristics, and formulation components can collectively influence interactions between plastics and microorganisms. However, the present results should be interpreted as differences in surface response rather than as evidence that one polymer type underwent complete biological degradation more readily than the other.

### 4.4 Integration of gravimetric, FTIR, and SEM evidence

A major strength of this study is the integration of complementary analytical approaches to characterize plastic surface responses to microalgal exposure. Gravimetric measurements, FTIR spectroscopy, and SEM observations provide different types of information and therefore allow the observed changes to be evaluated from complementary perspectives.

Gravimetric measurements alone cannot unequivocally demonstrate polymer biodegradation because apparent changes in mass may result from several processes, including removal or retention of surface associated material, residual biofilm, adsorbed extracellular polymeric substances, moisture, and analytical variability (Montazer et al., 2020). Small positive or negative weight changes may therefore occur without substantial modification of the polymer structure. For this reason, gravimetric measurements in the present study were interpreted as net mass changes and evaluated together with chemical and morphological observations.

FTIR spectroscopy provided evidence of changes in functional groups associated with the plastic surface. Alterations in carbonyl absorption bands, changes in aliphatic C H stretching intensity, and modifications within the fingerprint region indicate changes in surface associated chemical composition. These changes are consistent with physicochemical surface modification and may include oxidative processes, but they do not establish complete polymer degradation, depolymerization, or mineralization.

SEM analysis provided complementary information on surface morphology. Surface roughening, localized cracks, cavities, pits, fissures, and delamination like features were observed in several exposed samples. However, these features should not be interpreted as definitive indicators of biological erosion because polymer sample preparation, particularly ethanol dehydration and drying, may influence surface morphology. The use of identical preparation procedures for control and exposed samples supports comparative interpretation, while the possibility of preparation related artifacts remains an important limitation, particularly for thin LDPE films.

Taken together, the gravimetric, FTIR, and SEM results provide converging evidence of changes associated with the plastic surface following microalgal exposure. Nevertheless, the combined analytical evidence supports early stage surface modification rather than complete biodegradation or mineralization. Direct confirmation of polymer degradation would require additional measurements such as changes in molecular weight, carbon dioxide evolution, isotope tracing, or other approaches capable of demonstrating polymer conversion beyond surface associated alteration (Montazer et al., 2020; Mohanan et al., 2020).

The integration of multiple analytical approaches is increasingly recommended in plastic transformation studies because it reduces the risk of overinterpreting individual measurements and provides a more comprehensive assessment of physicochemical and morphological changes (Montazer et al., 2020; Mohanan et al., 2020).

### 4.5 Biological variability and interpretation of outliers

Considerable variability was observed among several biological replicates, including isolated observations with comparatively high or low weight change values. Such variability is possible in biological experiments involving microbial attachment and surface associated processes and should be interpreted together with the analytical characteristics of the measurements.

Microbial colonization of plastic surfaces can be heterogeneous because local differences in surface roughness, hydrophobicity, formulation characteristics, nutrient availability, and microbial attachment may influence the extent of surface associated biomass (Mohanan et al., 2020; Roy and Chakraborty, 2024). Consequently, biological replicates originating from the same commercial material may exhibit different levels of surface colonization and associated physicochemical responses even under identical incubation conditions.

The relatively high values observed for the blue PET sample in the *Scenedesmus dimorphus* treatment and the black LDPE mulch film in the *Chlorella vulgaris* treatment may therefore reflect biological and analytical variability rather than a consistent formulation specific response. These observations were retained in the primary analysis and evaluated through sensitivity analysis rather than being excluded solely because of their magnitude.

The statistical analysis accounted for non normality and variability through the use of the Aligned Rank Transform framework. The observed variation should therefore be considered when interpreting individual group means, particularly for groups with relatively high dispersion. Rather than treating isolated observations as evidence of enhanced degradation, the overall treatment patterns and complementary FTIR and SEM observations provide a more appropriate basis for interpretation.

### 4.6 Environmental implications

Plastic accumulation in aquatic and agricultural environments represents a major environmental challenge because conventional plastics can persist for extended periods and undergo physical and physicochemical transformation in natural environments. Consequently, biological processes capable of modifying plastic surfaces have attracted increasing scientific interest.

The findings of the present study indicate that freshwater microalgae can be associated with early stage physicochemical and morphological modification of commercial PET and LDPE surfaces under controlled laboratory conditions. The magnitude of these changes varied among plastic formulations, indicating that the interaction between microalgae and plastics may depend on both polymer characteristics and commercial formulation. However, the present results do not demonstrate complete biodegradation or mineralization of PET or LDPE.

Microalgal surface conditioning may nevertheless be relevant to subsequent microbial interactions in natural plastisphere communities. The production of extracellular polymeric substances and modification of local physicochemical conditions may influence the establishment of additional microorganisms on plastic surfaces (Brasil et al., 2017; Junior et al., 2020). In this context, microalgae may represent one component of a broader microbial succession rather than functioning as complete degraders of conventional plastics.

These observations may have implications for future biological plastic management strategies. Microalgae based systems could potentially be investigated as components of integrated microbial approaches for plastic surface conditioning or pretreatment. However, such applications require further validation under environmentally relevant conditions and with direct measurements of polymer transformation. Future studies combining microalgae with plastic associated bacterial communities, enzymatic analyses, molecular characterization, and long term experiments would help clarify whether microalgal surface modification contributes to subsequent polymer transformation.

### 4.7 Study limitations

Several limitations should be considered when interpreting the findings of this study. First, all experiments were conducted under controlled laboratory conditions and therefore cannot fully reproduce the environmental variability encountered in natural aquatic and terrestrial ecosystems. Factors such as ultraviolet radiation, fluctuating temperature, mechanical weathering, seasonal variation, and interactions with diverse microbial communities may influence plastic surface transformation under environmental conditions.

Second, only two freshwater microalgal species were evaluated during a 100 day incubation period. Although this period was sufficient to detect early physicochemical and morphological changes, longer exposure periods may be required to evaluate progressive surface modification and changes in microbial communities over time.

Third, the present study assessed plastic surface responses using gravimetric analysis, FTIR spectroscopy, and SEM observations. Although these complementary techniques provide converging evidence of surface modification, they do not directly demonstrate polymer mineralization. Measurements such as carbon dioxide evolution, isotope labeling, molecular weight determination using gel permeation chromatography, and thermal characterization by differential scanning calorimetry could provide additional evidence of polymer transformation and were beyond the scope of the present study.

Finally, the molecular mechanisms underlying microalgal plastic interactions were not directly investigated. Characterization of extracellular enzyme production, microbial community dynamics, transcriptomic responses, and metabolomic changes could provide further insight into the biological processes associated with the observed surface modifications.

Despite these limitations, the integration of gravimetric, spectroscopic, microscopic, and statistical analyses provides a comprehensive assessment of early stage microalgal plastic surface modification and establishes a basis for future mechanistic and environmentally relevant investigations.

## 5 Conclusion

This study evaluated the interaction of two freshwater microalgae, *Scenedesmus dimorphus* and *Chlorella vulgaris*, with commercially available PET and LDPE plastics containing different commercial formulations. By integrating gravimetric measurements, FTIR spectroscopy, and SEM observations, the study demonstrated measurable physicochemical and morphological changes at plastic surfaces following 100 days of microalgal exposure. However, the observed changes do not provide evidence of complete polymer degradation or mineralization.

The magnitude and nature of the observed surface modifications varied among the tested plastic formulations. Pigmented PET formulations generally exhibited greater FTIR associated changes than transparent PET, whereas black agricultural LDPE showed comparatively limited surface associated chemical modification. These differences highlight the importance of commercial formulation when evaluating plastic responses to microbial exposure, although the specific contribution of individual pigments or additives could not be established because their chemical identities and concentrations were not determined.

Both *Scenedesmus dimorphus* and *Chlorella vulgaris* were associated with surface modification of the tested plastics, although the magnitude and distribution of the observed responses varied among treatments and formulations. The combined gravimetric, FTIR, and SEM findings therefore support the interpretation that microalgal exposure can contribute to early stage physicochemical and morphological modification of plastic surfaces under controlled conditions.

The study indicates that microalgae may participate in the initial conditioning of conventional plastic surfaces, while the evidence does not support their characterization as complete degraders of PET or LDPE under the conditions tested. Future studies incorporating longer incubation periods, direct measurements of polymer molecular weight and mineralization, detailed characterization of commercial additives, and mixed microbial communities will be necessary to determine whether the observed surface modifications contribute to subsequent stages of polymer transformation.

## Data Availability

The original contributions presented in the study are included in the article/Supplementary material, further inquiries can be directed to the corresponding author.
