# Supplementary material for: Surface bio-erosion and structural alteration of colored and agricultural plastics induced by Scenedesmus dimorphus and Chlorella vulgaris
Source: Front Microbiol. 2026 Sep 23;17:1934915. doi: 10.3389/fmicb.2026.1934915 (PMC13642252; doi:10.3389/fmicb.2026.1934915)
Supplement: Supplementary file 1 [file Data_Sheet_1.PDF]

==

FRONTIERS IN MICROBIOLOGY STATISTICAL REVISION REPORT

==

MANUSCRIPT

Surface Bio-Erosion and Structural Alteration of Colored and Agricultural Plastics Induced by Scenedesmus dimorphus and Chlorella vulgaris

SAMPLE SIZE

Total observations: 108
Treatment levels: 3
Plastic levels: 6
Observations per experimental cell: 6

EXPERIMENTAL UNIT

The experimental unit was an individual plastic fragment. Each observation represents one independently weighed plastic fragment subjected to one Treatment × Plastic combination. There were six experimental units per combination, resulting in 108 observations across 18 experimental cells.

PRIMARY STATISTICAL METHOD

A two-factor ART ANOVA was used as the primary inferential analysis because the data showed substantial departures from normality and heterogeneity of variance across experimental cells.

==

ART ANOVA

==

```
               Term Df Df.res   Sum Sq Sum Sq.res   F value
Pr(>F)
1         Treatment  2     90 26404.06   52720.33 22.537462
0.00000001161582
2           Plastic  5     90 10518.69   75704.33  2.501000
0.03617030853895
3 Treatment:Plastic 10     90 19110.94   45146.08  3.809821
0.00025958556354
  partial_eta_squared eta_interpretation significance
1           0.3337031              Large          ***
2           0.1219940             Medium            *
3           0.2974141              Large          ***
```

==

SHAPIRO-WILK NORMALITY TESTS

==

```
# A tibble: 18 × 6
   Treatment   Plastic  variable              W   p_value
Normality_assumption
   <fct>       <fct>    <chr>             <dbl>     <dbl> <chr>
 1 Control     Blue     WeightLoss_percent 0.871 0.232     Not rejected
 2 Control     Green    WeightLoss_percent 0.683 0.00404   Violated
```

3	Control	Gray	WeightLoss_percent	0.817	0.0824	Not rejected
4	Control	Black	WeightLoss_percent	0.862	0.196	Not rejected
5	Control	White	WeightLoss_percent	0.926	0.553	Not rejected
6	Control	PetWhite	WeightLoss_percent	0.867	0.213	Not rejected
7	Scenedesmus	Blue	WeightLoss_percent	0.528	0.0000564	Violated
8	Scenedesmus	Green	WeightLoss_percent	0.973	0.910	Not rejected
9	Scenedesmus	Gray	WeightLoss_percent	0.717	0.00922	Violated
10	Scenedesmus	Black	WeightLoss_percent	0.941	0.665	Not rejected
11	Scenedesmus	White	WeightLoss_percent	0.786	0.0434	Violated
12	Scenedesmus	PetWhite	WeightLoss_percent	0.901	0.379	Not rejected
13	Chlorella	Blue	WeightLoss_percent	0.948	0.728	Not rejected
14	Chlorella	Green	WeightLoss_percent	0.719	0.00964	Violated
15	Chlorella	Gray	WeightLoss_percent	0.924	0.532	Not rejected
16	Chlorella	Black	WeightLoss_percent	0.668	0.00281	Violated
17	Chlorella	White	WeightLoss_percent	0.784	0.0420	Violated
18	Chlorella	PetWhite	WeightLoss_percent	0.924	0.536	Not rejected

==

BROWN-FORSYTHE / LEVENE TEST

==

	Df	F value	Pr(>F)
group	17	1.860441	0.03212403
	90	NA	NA

==

ALL 153 TUKEY-ADJUSTED CELL COMPARISONS

==

	contrast	estimate	SE	df
1	Control,Black - Scenedesmus,White	-79.25000000	15.57818	90
2	Chlorella,Gray - Control,Black	70.16666667	15.57818	90
3	Control,Black - Control,Green	-65.16666667	15.57818	90
4	Control,Black - Scenedesmus,Green	-62.83333333	15.57818	90
5	Control,Black - Scenedesmus,Blue	-62.16666667	15.57818	90
6	Control,White - Scenedesmus,White	-57.58333333	15.57818	90
7	Control,Black - Scenedesmus,Gray	-54.58333333	15.57818	90
8	Chlorella,Black - Scenedesmus,White	-54.50000000	15.57818	90
9	Chlorella,PetWhite - Control,Black	53.75000000	15.57818	90
10	Chlorella,White - Control,Black	53.58333333	15.57818	90
11	Chlorella,Blue - Control,Black	52.16666667	15.57818	90
12	Scenedesmus,Black - Scenedesmus,White	-50.16666667	15.57818	90
13	Chlorella,Gray - Control,White	48.50000000	15.57818	90
14	Chlorella,Black - Chlorella,Gray	-45.41666667	15.57818	90
15	Control,Black - Control,PetWhite	-44.25000000	15.57818	90
16	Control,Green - Control,White	43.50000000	15.57818	90
17	Control,Black - Control,Blue	-43.00000000	15.57818	90
18	Control,Black - Scenedesmus,PetWhite	-41.66666667	15.57818	90
19	Control,Black - Control,Gray	-41.58333333	15.57818	90
20	Control,White - Scenedesmus,Green	-41.16666667	15.57818	90
21	Chlorella,Gray - Scenedesmus,Black	41.08333333	15.57818	90
22	Control,White - Scenedesmus,Blue	-40.50000000	15.57818	90

23	Chlorella,Black - Control,Green	-40.41666667	15.57818	90
24	Chlorella,Green - Control,Black	40.33333333	15.57818	90
25	Chlorella,Green - Scenedesmus,White	-38.91666667	15.57818	90
26	Chlorella,Black - Scenedesmus,Green	-38.08333333	15.57818	90
27	Control,Gray - Scenedesmus,White	-37.66666667	15.57818	90
28	Scenedesmus,PetWhite - Scenedesmus,White	-37.58333333	15.57818	90
29	Chlorella,Black - Scenedesmus,Blue	-37.41666667	15.57818	90
30	Control,Blue - Scenedesmus,White	-36.25000000	15.57818	90
31	Control,Green - Scenedesmus,Black	36.08333333	15.57818	90
32	Control,PetWhite - Scenedesmus,White	-35.00000000	15.57818	90
33	Scenedesmus,Black - Scenedesmus,Green	-33.75000000	15.57818	90
34	Scenedesmus,Black - Scenedesmus,Blue	-33.08333333	15.57818	90
35	Control,White - Scenedesmus,Gray	-32.91666667	15.57818	90
36	Chlorella,PetWhite - Control,White	32.08333333	15.57818	90
37	Chlorella,White - Control,White	31.91666667	15.57818	90
38	Chlorella,Blue - Control,White	30.50000000	15.57818	90
39	Chlorella,Black - Scenedesmus,Gray	-29.83333333	15.57818	90
40	Chlorella,Gray - Chlorella,Green	29.83333333	15.57818	90
41	Control,Black - Scenedesmus,Black	-29.08333333	15.57818	90
42	Chlorella,Black - Chlorella,PetWhite	-29.00000000	15.57818	90
43	Chlorella,Black - Chlorella,White	-28.83333333	15.57818	90
44	Chlorella,Gray - Control,Gray	28.58333333	15.57818	90
45	Chlorella,Gray - Scenedesmus,PetWhite	28.50000000	15.57818	90
46	Chlorella,Black - Chlorella,Blue	-27.41666667	15.57818	90
47	Chlorella,Gray - Control,Blue	27.16666667	15.57818	90
48	Chlorella,Blue - Scenedesmus,White	-27.08333333	15.57818	90
49	Chlorella,Gray - Control,PetWhite	25.91666667	15.57818	90
50	Chlorella,White - Scenedesmus,White	-25.66666667	15.57818	90
51	Chlorella,PetWhite - Scenedesmus,White	-25.50000000	15.57818	90
52	Scenedesmus,Black - Scenedesmus,Gray	-25.50000000	15.57818	90
53	Chlorella,Green - Control,Green	-24.83333333	15.57818	90
54	Chlorella,Black - Control,Black	24.75000000	15.57818	90
55	Chlorella,PetWhite - Scenedesmus,Black	24.66666667	15.57818	90
56	Scenedesmus,Gray - Scenedesmus,White	-24.66666667	15.57818	90
57	Chlorella,White - Scenedesmus,Black	24.50000000	15.57818	90
58	Control,Gray - Control,Green	-23.58333333	15.57818	90
59	Control,Green - Scenedesmus,PetWhite	23.50000000	15.57818	90
60	Chlorella,Blue - Scenedesmus,Black	23.08333333	15.57818	90
61	Control,PetWhite - Control,White	22.58333333	15.57818	90
62	Chlorella,Green - Scenedesmus,Green	-22.50000000	15.57818	90
63	Control,Blue - Control,Green	-22.16666667	15.57818	90
64	Chlorella,Green - Scenedesmus,Blue	-21.83333333	15.57818	90
65	Control,Black - Control,White	-21.66666667	15.57818	90
66	Control,Blue - Control,White	21.33333333	15.57818	90
67	Control,Gray - Scenedesmus,Green	-21.25000000	15.57818	90
68	Scenedesmus,Green - Scenedesmus,PetWhite	21.16666667	15.57818	90
69	Control,Green - Control,PetWhite	20.91666667	15.57818	90
70	Control,Gray - Scenedesmus,Blue	-20.58333333	15.57818	90
71	Scenedesmus,Blue - Scenedesmus,PetWhite	20.50000000	15.57818	90
72	Control,White - Scenedesmus,PetWhite	-20.00000000	15.57818	90
73	Control,Gray - Control,White	19.91666667	15.57818	90
74	Control,Blue - Scenedesmus,Green	-19.83333333	15.57818	90
75	Chlorella,Black - Control,PetWhite	-19.50000000	15.57818	90
76	Control,Blue - Scenedesmus,Blue	-19.16666667	15.57818	90

77	Chlorella,Green - Control,White	18.66666667	15.57818	90
78	Control,PetWhite - Scenedesmus,Green	-18.58333333	15.57818	90
79	Chlorella,Black - Control,Blue	-18.25000000	15.57818	90
80	Chlorella,Blue - Chlorella,Gray	-18.00000000	15.57818	90
81	Control,PetWhite - Scenedesmus,Blue	-17.91666667	15.57818	90
82	Scenedesmus,Blue - Scenedesmus,White	-17.08333333	15.57818	90
83	Chlorella,Black - Scenedesmus,PetWhite	-16.91666667	15.57818	90
84	Chlorella,Black - Control,Gray	-16.83333333	15.57818	90
85	Chlorella,Gray - Chlorella,White	16.58333333	15.57818	90
86	Chlorella,Gray - Chlorella,PetWhite	16.41666667	15.57818	90
87	Scenedesmus,Green - Scenedesmus,White	-16.41666667	15.57818	90
88	Chlorella,Black - Chlorella,Green	-15.58333333	15.57818	90
89	Chlorella,Gray - Scenedesmus,Gray	15.58333333	15.57818	90
90	Control,PetWhite - Scenedesmus,Black	15.16666667	15.57818	90
91	Chlorella,Green - Scenedesmus,Gray	-14.25000000	15.57818	90
92	Control,Green - Scenedesmus,White	-14.08333333	15.57818	90
93	Control,Blue - Scenedesmus,Black	13.91666667	15.57818	90
94	Chlorella,Green - Chlorella,PetWhite	-13.41666667	15.57818	90
95	Chlorella,Green - Chlorella,White	-13.25000000	15.57818	90
96	Chlorella,Blue - Control,Green	-13.00000000	15.57818	90
97	Control,Gray - Scenedesmus,Gray	-13.00000000	15.57818	90
98	Scenedesmus,Gray - Scenedesmus,PetWhite	12.91666667	15.57818	90
99	Scenedesmus,Black - Scenedesmus,PetWhite	-12.58333333	15.57818	90
100	Control,Gray - Scenedesmus,Black	12.50000000	15.57818	90
101	Chlorella,PetWhite - Control,Gray	12.16666667	15.57818	90
102	Chlorella,PetWhite - Scenedesmus,PetWhite	12.08333333	15.57818	90
103	Chlorella,White - Control,Gray	12.00000000	15.57818	90
104	Chlorella,White - Scenedesmus,PetWhite	11.91666667	15.57818	90
105	Chlorella,Blue - Chlorella,Green	11.83333333	15.57818	90
106	Chlorella,White - Control,Green	-11.58333333	15.57818	90
107	Control,Blue - Scenedesmus,Gray	-11.58333333	15.57818	90
108	Chlorella,PetWhite - Control,Green	-11.41666667	15.57818	90
109	Chlorella,Green - Scenedesmus,Black	11.25000000	15.57818	90
110	Chlorella,PetWhite - Control,Blue	10.75000000	15.57818	90
111	Chlorella,Blue - Scenedesmus,Green	-10.66666667	15.57818	90
112	Chlorella,Blue - Control,Gray	10.58333333	15.57818	90
113	Chlorella,White - Control,Blue	10.58333333	15.57818	90
114	Control,Green - Scenedesmus,Gray	10.58333333	15.57818	90
115	Chlorella,Blue - Scenedesmus,PetWhite	10.50000000	15.57818	90
116	Control,PetWhite - Scenedesmus,Gray	-10.33333333	15.57818	90
117	Chlorella,Blue - Scenedesmus,Blue	-10.00000000	15.57818	90
118	Chlorella,PetWhite - Control,PetWhite	9.50000000	15.57818	90
119	Chlorella,White - Control,PetWhite	9.33333333	15.57818	90
120	Chlorella,White - Scenedesmus,Green	-9.25000000	15.57818	90
121	Chlorella,Blue - Control,Blue	9.16666667	15.57818	90
122	Chlorella,Gray - Scenedesmus,White	-9.08333333	15.57818	90
123	Chlorella,PetWhite - Scenedesmus,Green	-9.08333333	15.57818	90
124	Chlorella,White - Scenedesmus,Blue	-8.58333333	15.57818	90
125	Chlorella,PetWhite - Scenedesmus,Blue	-8.41666667	15.57818	90
126	Scenedesmus,Gray - Scenedesmus,Green	-8.25000000	15.57818	90
127	Chlorella,Gray - Scenedesmus,Blue	8.00000000	15.57818	90
128	Chlorella,Blue - Control,PetWhite	7.91666667	15.57818	90
129	Scenedesmus,Blue - Scenedesmus,Gray	7.58333333	15.57818	90
130	Control,White - Scenedesmus,Black	-7.41666667	15.57818	90

131	Chlorella,Gray - Scenedesmus,Green	7.33333333	15.57818	90
132	Chlorella,Gray - Control,Green	5.00000000	15.57818	90
133	Chlorella,Black - Control,White	3.08333333	15.57818	90
134	Chlorella,Black - Scenedesmus,Black	-4.33333333	15.57818	90
135	Chlorella,Blue - Chlorella,PetWhite	-1.58333333	15.57818	90
136	Chlorella,Blue - Chlorella,White	-1.41666667	15.57818	90
137	Chlorella,Blue - Scenedesmus,Gray	-2.41666667	15.57818	90
138	Chlorella,Green - Control,Blue	-2.66666667	15.57818	90
139	Chlorella,Green - Control,Gray	-1.25000000	15.57818	90
140	Chlorella,Green - Control,PetWhite	-3.91666667	15.57818	90
141	Chlorella,Green - Scenedesmus,PetWhite	-1.33333333	15.57818	90
142	Chlorella,PetWhite - Chlorella,White	0.16666667	15.57818	90
143	Chlorella,PetWhite - Scenedesmus,Gray	-0.83333333	15.57818	90
144	Chlorella,White - Scenedesmus,Gray	-1.00000000	15.57818	90
145	Control,Blue - Control,Gray	1.41666667	15.57818	90
146	Control,Blue - Control,PetWhite	-1.25000000	15.57818	90
147	Control,Blue - Scenedesmus,PetWhite	1.33333333	15.57818	90
148	Control,Gray - Control,PetWhite	-2.66666667	15.57818	90
149	Control,Gray - Scenedesmus,PetWhite	-0.08333333	15.57818	90
150	Control,Green - Scenedesmus,Blue	3.00000000	15.57818	90
151	Control,Green - Scenedesmus,Green	2.33333333	15.57818	90
152	Control,PetWhite - Scenedesmus,PetWhite	2.58333333	15.57818	90
153	Scenedesmus,Blue - Scenedesmus,Green	-0.66666667	15.57818	90

	lower.CL	upper.CL	t.ratio	p.value	r_effect
1	-135.211244	-23.288756	-5.087244479	0.0002696136	0.4725831799
2	14.205422	126.127911	4.504163882	0.0024997174	0.4288950686
3	-121.127911	-9.205422	-4.183202085	0.0077580696	0.4034653752
4	-118.794578	-6.872089	-4.033419913	0.0128122001	0.3912651901
5	-118.127911	-6.205422	-3.990625007	0.0147362893	0.3877408009
6	-113.544578	-1.622089	-3.696410026	0.0368758449	0.3630507318
7	-110.544578	1.377911	-3.503832948	0.0641781978	0.3464612997
8	-110.461244	1.461244	-3.498483585	0.0651366434	0.3459957158
9	-2.211244	109.711244	3.450339316	0.0743258930	0.3417939713
10	-2.377911	109.544578	3.439640589	0.0765106767	0.3408574488
11	-3.794578	108.127911	3.348701413	0.0973504289	0.3328561287
12	-106.127911	5.794578	-3.220316695	0.1344768633	0.3214368551
13	-7.461244	104.461244	3.113329429	0.1732205422	0.3118122234
14	-101.377911	10.544578	-2.915402988	0.2655169261	0.2937523956
15	-100.211244	11.711244	-2.840511902	0.3075074327	0.2868347927
16	-12.461244	99.461244	2.792367632	0.3364338396	0.2823638696
17	-98.961244	12.961244	-2.760271452	0.3565052153	0.2793729782
18	-97.627911	14.294578	-2.674681640	0.4127950452	0.2713575617
19	-97.544578	14.377911	-2.669332277	0.4164332198	0.2708546983
20	-97.127911	14.794578	-2.642585460	0.4348096825	0.2683370596
21	-14.877911	97.044578	2.637236097	0.4385202445	0.2678328695
22	-96.461244	15.461244	-2.599790554	0.4647895224	0.2642973908
23	-96.377911	15.544578	-2.594441191	0.4685809492	0.2637914477
24	-15.627911	96.294578	2.589091828	0.4723811345	0.2632852867
25	-94.877911	17.044578	-2.498152652	0.5380071695	0.2546475111
26	-94.044578	17.877911	-2.444659019	0.5770713303	0.2495376955
27	-93.627911	18.294578	-2.417912203	0.5965821820	0.2469749193
28	-93.544578	18.377911	-2.412562839	0.6004764133	0.2464617397
29	-93.377911	18.544578	-2.401864113	0.6082537678	0.2454347585
30	-92.211244	19.711244	-2.326973027	0.6620269578	0.2382228791

31	-19.877911	92.044578	2.316274300	0.6695750034	0.2371893548
32	-90.961244	20.961244	-2.246732578	0.7174508383	0.2304519204
33	-89.711244	22.211244	-2.166492128	0.7693570967	0.2226366197
34	-89.044578	22.877911	-2.123697222	0.7952051165	0.2184507353
35	-88.877911	23.044578	-2.112998496	0.8014428720	0.2174023674
36	-23.877911	88.044578	2.059504863	0.8311865825	0.2121492925
37	-24.044578	87.877911	2.048806136	0.8368342833	0.2110964511
38	-25.461244	86.461244	1.957866960	0.8805352563	0.2021179173
39	-85.794578	26.127911	-1.915072054	0.8983487722	0.1978748744
40	-26.127911	85.794578	1.915072054	0.8983487722	0.1978748744
41	-85.044578	26.877911	-1.866927785	0.9162579417	0.1930881311
42	-84.961244	26.961244	-1.861578421	0.9181095644	0.1925554119
43	-84.794578	27.127911	-1.850879695	0.9217306560	0.1914894631
44	-27.377911	84.544578	1.834831605	0.9269583060	0.1898892696
45	-27.461244	84.461244	1.829482242	0.9286468770	0.1893555346
46	-83.377911	28.544578	-1.759940519	0.9482110001	0.1824018488
47	-28.794578	83.127911	1.743892429	0.9521181361	0.1807932169
48	-83.044578	28.877911	-1.738543066	0.9533719689	0.1802566829
49	-30.044578	81.877911	1.663651980	0.9685145471	0.1727284985
50	-81.627911	30.294578	-1.647603890	0.9712100097	0.1711113224
51	-81.461244	30.461244	-1.636905164	0.9729071920	0.1700324352
52	-81.461244	30.461244	-1.636905164	0.9729071920	0.1700324352
53	-80.794578	31.127911	-1.594110257	0.9789432944	0.1657108031
54	-31.211244	80.711244	1.588760894	0.9796175569	0.1651699218
55	-31.294578	80.627911	1.583411531	0.9802748865	0.1646288915
56	-80.627911	31.294578	-1.583411531	0.9802748865	0.1646288915
57	-31.461244	80.461244	1.572712804	0.9815398328	0.1635463853
58	-79.544578	32.377911	-1.513869808	0.9874024654	0.1575821296
59	-32.461244	79.461244	1.508520445	0.9878510893	0.1570390581
60	-32.877911	79.044578	1.481773629	0.9899057123	0.1543215720
61	-33.377911	78.544578	1.449677449	0.9919900718	0.1510559647
62	-78.461244	33.461244	-1.444328086	0.9923004958	0.1505112127
63	-78.127911	33.794578	-1.422930632	0.9934452836	0.1483308376
64	-77.794578	34.127911	-1.401533179	0.9944459610	0.1461482973
65	-77.627911	34.294578	-1.390834453	0.9948966625	0.1450562243
66	-34.627911	77.294578	1.369437000	0.9957071844	0.1428704906
67	-77.211244	34.711244	-1.364087636	0.9958920558	0.1423237291
68	-34.794578	77.127911	1.358738273	0.9960702136	0.1417768373
69	-35.044578	76.877911	1.342690183	0.9965662831	0.1401353847
70	-76.544578	35.377911	-1.321292730	0.9971448355	0.1379449847
71	-35.461244	76.461244	1.315943367	0.9972758282	0.1373970670
72	-75.961244	35.961244	-1.283847187	0.9979595948	0.1341069277
73	-36.044578	75.877911	1.278497824	0.9980579390	0.1335581367
74	-75.794578	36.127911	-1.273148461	0.9981522058	0.1330092228
75	-75.461244	36.461244	-1.251751008	0.9984911890	0.1308123477
76	-75.127911	36.794578	-1.230353554	0.9987753143	0.1286135439
77	-37.294578	74.627911	1.198257375	0.9991147510	0.1253117880
78	-74.544578	37.377911	-1.192908011	0.9991625362	0.1247610873
79	-74.211244	37.711244	-1.171510558	0.9993319890	0.1225571359
80	-73.961244	37.961244	-1.155462468	0.9994386542	0.1209029797
81	-73.877911	38.044578	-1.150113105	0.9994707355	0.1203513698
82	-73.044578	38.877911	-1.096619472	0.9997133120	0.1148292174
83	-72.877911	39.044578	-1.085920746	0.9997478057	0.1137234914
84	-72.794578	39.127911	-1.080571383	0.9997636347	0.1131704697

85	-39.377911	72.544578	1.064523293	0.9998059792	0.1115107752
86	-39.544578	72.377911	1.053824566	0.9998303303	0.1104037928
87	-72.377911	39.544578	-1.053824566	0.9998303303	0.1104037928
88	-71.544578	40.377911	-1.000330933	0.9999159154	0.1048627935
89	-40.377911	71.544578	1.000330933	0.9999159154	0.1048627935
90	-40.794578	71.127911	0.973584117	0.9999420232	0.1020885942
91	-70.211244	41.711244	-0.914741121	0.9999757352	0.0959770519
92	-70.044578	41.877911	-0.904042394	0.9999794612	0.0948646746
93	-42.044578	69.877911	0.893343668	0.9999826620	0.0937519418
94	-69.377911	42.544578	-0.861247488	0.9999897445	0.0904116509
95	-69.211244	42.711244	-0.850548762	0.9999914412	0.0892975366
96	-68.961244	42.961244	-0.834500672	0.9999935118	0.0876257376
97	-68.961244	42.961244	-0.834500672	0.9999935118	0.0876257376
98	-43.044578	68.877911	0.829151308	0.9999940932	0.0870683060
99	-68.544578	43.377911	-0.807753855	0.9999959749	0.0848377667
100	-43.461244	68.461244	0.802404492	0.9999963504	0.0842799312
101	-43.794578	68.127911	0.781007039	0.9999975543	0.0820478018
102	-43.877911	68.044578	0.775657676	0.9999977920	0.0814895751
103	-43.961244	67.961244	0.770308312	0.9999980084	0.0809312717
104	-44.044578	67.877911	0.764958949	0.9999982052	0.0803728922
105	-44.127911	67.794578	0.759609586	0.9999983841	0.0798144370
106	-67.544578	44.377911	-0.743561496	0.9999988273	0.0781386223
107	-67.544578	44.377911	-0.743561496	0.9999988273	0.0781386223
108	-67.377911	44.544578	-0.732862769	0.9999990575	0.0770210436
109	-44.711244	67.211244	0.722164043	0.9999992455	0.0759031745
110	-45.211244	66.711244	0.690067863	0.9999996225	0.0725478659
111	-66.627911	45.294578	-0.684718500	0.9999996650	0.0719884052
112	-45.377911	66.544578	0.679369137	0.9999997030	0.0714288765
113	-45.377911	66.544578	0.679369137	0.9999997030	0.0714288765
114	-45.377911	66.544578	0.679369137	0.9999997030	0.0714288765
115	-45.461244	66.461244	0.674019773	0.9999997369	0.0708692803
116	-66.294578	45.627911	-0.663321047	0.9999997944	0.0697498877
117	-65.961244	45.961244	-0.641923594	0.9999998762	0.0675103162
118	-46.461244	65.461244	0.609827414	0.9999999444	0.0641490549
119	-46.627911	65.294578	0.599128687	0.9999999579	0.0630281434
120	-65.211244	46.711244	-0.593779324	0.9999999634	0.0624675980
121	-46.794578	65.127911	0.588429961	0.9999999683	0.0619069934
122	-65.044578	46.877911	-0.583080598	0.9999999725	0.0613463302
123	-65.044578	46.877911	-0.583080598	0.9999999725	0.0613463302
124	-64.544578	47.377911	-0.550984418	0.9999999888	0.0579811499
125	-64.377911	47.544578	-0.540285691	0.9999999918	0.0568589780
126	-64.211244	47.711244	-0.529586965	0.9999999940	0.0557365906
127	-47.961244	63.961244	0.513538875	0.9999999964	0.0540526146
128	-48.044578	63.877911	0.508189512	0.9999999969	0.0534911860
129	-48.377911	63.544578	0.486792058	0.9999999985	0.0512449697
130	-63.377911	48.544578	-0.476093332	0.9999999989	0.0501215677
131	-48.627911	63.294578	0.470743969	0.9999999991	0.0495597951
132	-50.961244	60.961244	0.320961797	1.0000000000	0.0338129979
133	-52.877911	59.044578	0.197926441	1.0000000000	0.0208587396
134	-60.294578	51.627911	-0.278166891	1.0000000000	0.0293087685
135	-57.544578	54.377911	-0.101637902	1.0000000000	0.0107129608
136	-57.377911	54.544578	-0.090939176	1.0000000000	0.0095853904
137	-58.377911	53.544578	-0.155131535	1.0000000000	0.0163501138
138	-58.627911	53.294578	-0.171179625	1.0000000000	0.0180409801

139	-57.211244	54.711244	-0.080240449	1.0000000000	0.0084577835
140	-59.877911	52.044578	-0.251420074	1.0000000000	0.0264927008
141	-57.294578	54.627911	-0.085589812	1.0000000000	0.0090215912
142	-55.794578	56.127911	0.010698727	1.0000000000	0.0011277441
143	-56.794578	55.127911	-0.053493633	1.0000000000	0.0056386344
144	-56.961244	54.961244	-0.064192359	1.0000000000	0.0067663139
145	-54.544578	57.377911	0.090939176	1.0000000000	0.0095853904
146	-57.211244	54.711244	-0.080240449	1.0000000000	0.0084577835
147	-54.627911	57.294578	0.085589812	1.0000000000	0.0090215912
148	-58.627911	53.294578	-0.171179625	1.0000000000	0.0180409801
149	-56.044578	55.877911	-0.005349363	1.0000000000	0.0005638723
150	-52.961244	58.961244	0.192577078	1.0000000000	0.0202952253
151	-53.627911	58.294578	0.149782172	1.0000000000	0.0157864597
152	-53.377911	58.544578	0.165830262	1.0000000000	0.0174773745
153	-56.627911	55.294578	-0.042794906	1.0000000000	0.0045109333

	r_interpretation	significance
1	Medium	***
2	Medium	**
3	Medium	**
4	Medium	*
5	Medium	*
6	Medium	*
7	Medium	ns
8	Medium	ns
9	Medium	ns
10	Medium	ns
11	Medium	ns
12	Medium	ns
13	Medium	ns
14	Small	ns
15	Small	ns
16	Small	ns
17	Small	ns
18	Small	ns
19	Small	ns
20	Small	ns
21	Small	ns
22	Small	ns
23	Small	ns
24	Small	ns
25	Small	ns
26	Small	ns
27	Small	ns
28	Small	ns
29	Small	ns
30	Small	ns
31	Small	ns
32	Small	ns
33	Small	ns
34	Small	ns
35	Small	ns
36	Small	ns
37	Small	ns
38	Small	ns

39	Small	ns
40	Small	ns
41	Small	ns
42	Small	ns
43	Small	ns
44	Small	ns
45	Small	ns
46	Small	ns
47	Small	ns
48	Small	ns
49	Small	ns
50	Small	ns
51	Small	ns
52	Small	ns
53	Small	ns
54	Small	ns
55	Small	ns
56	Small	ns
57	Small	ns
58	Small	ns
59	Small	ns
60	Small	ns
61	Small	ns
62	Small	ns
63	Small	ns
64	Small	ns
65	Small	ns
66	Small	ns
67	Small	ns
68	Small	ns
69	Small	ns
70	Small	ns
71	Small	ns
72	Small	ns
73	Small	ns
74	Small	ns
75	Small	ns
76	Small	ns
77	Small	ns
78	Small	ns
79	Small	ns
80	Small	ns
81	Small	ns
82	Small	ns
83	Small	ns
84	Small	ns
85	Small	ns
86	Small	ns
87	Small	ns
88	Small	ns
89	Small	ns
90	Small	ns
91	Very small	ns
92	Very small	ns

93	Very small	ns
94	Very small	ns
95	Very small	ns
96	Very small	ns
97	Very small	ns
98	Very small	ns
99	Very small	ns
100	Very small	ns
101	Very small	ns
102	Very small	ns
103	Very small	ns
104	Very small	ns
105	Very small	ns
106	Very small	ns
107	Very small	ns
108	Very small	ns
109	Very small	ns
110	Very small	ns
111	Very small	ns
112	Very small	ns
113	Very small	ns
114	Very small	ns
115	Very small	ns
116	Very small	ns
117	Very small	ns
118	Very small	ns
119	Very small	ns
120	Very small	ns
121	Very small	ns
122	Very small	ns
123	Very small	ns
124	Very small	ns
125	Very small	ns
126	Very small	ns
127	Very small	ns
128	Very small	ns
129	Very small	ns
130	Very small	ns
131	Very small	ns
132	Very small	ns
133	Very small	ns
134	Very small	ns
135	Very small	ns
136	Very small	ns
137	Very small	ns
138	Very small	ns
139	Very small	ns
140	Very small	ns
141	Very small	ns
142	Very small	ns
143	Very small	ns
144	Very small	ns
145	Very small	ns
146	Very small	ns

```
147       Very small          ns
148       Very small          ns
149       Very small          ns
150       Very small          ns
151       Very small          ns
152       Very small          ns
153       Very small          ns
```

```
============================================================
SIGNIFICANT CELL COMPARISONS
============================================================

                           contrast  estimate       SE df   lower.CL
upper.CL
1 Control,Black - Scenedesmus,White -79.25000 15.57818 90 -135.21124 -
23.288756
2    Chlorella,Gray - Control,Black  70.16667 15.57818 90   14.20542
126.127911
3     Control,Black - Control,Green -65.16667 15.57818 90 -121.12791  -
9.205422
4 Control,Black - Scenedesmus,Green -62.83333 15.57818 90 -118.79458  -
6.872089
5  Control,Black - Scenedesmus,Blue -62.16667 15.57818 90 -118.12791  -
6.205422
6 Control,White - Scenedesmus,White -57.58333 15.57818 90 -113.54458  -
1.622089
    t.ratio      p.value  r_effect r_interpretation significance
1 -5.087244 0.0002696136 0.4725832           Medium          ***
2  4.504164 0.0024997174 0.4288951           Medium           **
3 -4.183202 0.0077580696 0.4034654           Medium           **
4 -4.033420 0.0128122001 0.3912652           Medium            *
5 -3.990625 0.0147362893 0.3877408           Medium            *
6 -3.696410 0.0368758449 0.3630507           Medium            *
```

```
============================================================
ALL 45 TRUE INTERACTION CONTRASTS
Difference-of-differences
============================================================

        Treatment_pairwise Plastic_pairwise   estimate       SE df
lower.CL
1      Control - Chlorella    Green - Black  85.250000 18.28703 90
23.777524
2      Control - Chlorella     Blue - Black  77.833333 18.28703 90
16.360857
3    Control - Scenedesmus     Gray - Black  69.583333 18.28703 90
8.110857
4    Control - Scenedesmus    Green - Black  66.916667 18.28703 90
5.444190
5    Control - Scenedesmus     Blue - Black  63.833333 18.28703 90
2.360857
```

```
6      Control - Chlorella     Gray - Black  63.416667 18.28703 90
1.944190
7    Control - Scenedesmus Black - PetWhite -59.833333 18.28703 90 -
121.305810
8      Control - Chlorella Black - PetWhite -58.500000 18.28703 90 -
119.972476
9      Control - Chlorella    Black - White -58.000000 18.28703 90 -
119.472476
10   Control - Scenedesmus     Gray - White  43.250000 18.28703 90  -
18.222476
11   Control - Scenedesmus    Green - White  40.583333 18.28703 90  -
20.889143
12 Scenedesmus - Chlorella     Gray - White -37.833333 18.28703 90  -
99.305810
13   Control - Scenedesmus     Blue - White  37.500000 18.28703 90  -
23.972476
14   Control - Scenedesmus White - PetWhite -33.500000 18.28703 90  -
94.972476
15 Scenedesmus - Chlorella White - PetWhite  33.000000 18.28703 90  -
28.472476
16 Scenedesmus - Chlorella    Black - White -31.666667 18.28703 90  -
93.139143
17     Control - Chlorella    Green - White  27.250000 18.28703 90  -
34.222476
18     Control - Chlorella Green - PetWhite  26.750000 18.28703 90  -
34.722476
19   Control - Scenedesmus    Black - White -26.333333 18.28703 90  -
87.805810
20 Scenedesmus - Chlorella     Green - Gray  24.500000 18.28703 90  -
36.972476
21     Control - Chlorella     Green - Gray  21.833333 18.28703 90  -
39.639143
22 Scenedesmus - Chlorella      Blue - Gray  20.166667 18.28703 90  -
41.305810
23     Control - Chlorella     Blue - White  19.833333 18.28703 90  -
41.639143
24 Scenedesmus - Chlorella Green - PetWhite  19.666667 18.28703 90  -
41.805810
25     Control - Chlorella  Blue - PetWhite  19.333333 18.28703 90  -
42.139143
26 Scenedesmus - Chlorella    Green - Black  18.333333 18.28703 90  -
43.139143
27 Scenedesmus - Chlorella     Blue - White -17.666667 18.28703 90  -
79.139143
28 Scenedesmus - Chlorella  Blue - PetWhite  15.333333 18.28703 90  -
46.139143
29     Control - Chlorella      Blue - Gray  14.416667 18.28703 90  -
47.055810
30 Scenedesmus - Chlorella     Blue - Black  14.000000 18.28703 90  -
47.472476
31 Scenedesmus - Chlorella    Green - White -13.333333 18.28703 90  -
74.805810
32   Control - Scenedesmus     Blue - Green  -3.083333 18.28703 90  -
64.555810
```

```
33     Control - Chlorella     Blue - Green  -7.416667 18.28703 90  -
68.889143
34 Scenedesmus - Chlorella     Blue - Green  -4.333333 18.28703 90  -
65.805810
35   Control - Scenedesmus      Blue - Gray  -5.750000 18.28703 90  -
67.222476
36   Control - Scenedesmus  Blue - PetWhite   4.000000 18.28703 90  -
57.472476
37   Control - Scenedesmus     Green - Gray  -2.666667 18.28703 90  -
64.139143
38   Control - Scenedesmus Green - PetWhite   7.083333 18.28703 90  -
54.389143
39 Scenedesmus - Chlorella     Gray - Black  -6.166667 18.28703 90  -
67.639143
40     Control - Chlorella     Gray - White   5.416667 18.28703 90  -
56.055810
41   Control - Scenedesmus  Gray - PetWhite   9.750000 18.28703 90  -
51.722476
42     Control - Chlorella  Gray - PetWhite   4.916667 18.28703 90  -
56.555810
43 Scenedesmus - Chlorella  Gray - PetWhite  -4.833333 18.28703 90  -
66.305810
44 Scenedesmus - Chlorella Black - PetWhite   1.333333 18.28703 90  -
60.139143
45     Control - Chlorella White - PetWhite  -0.500000 18.28703 90  -
61.972476
     upper.CL     t.ratio     p.value    r_effect r_interpretation
significance
1  146.722476  4.66177418 0.000486853 0.441023952           Medium
***
2  139.305810  4.25620439 0.002287186 0.409335061           Medium
**
3  131.055810  3.80506495 0.011550811 0.372261883           Medium
*
4  128.389143  3.65924210 0.018991371 0.359875073           Medium
*
5  125.305810  3.49063443 0.033134803 0.345312099           Medium
*
6  124.889143  3.46784961 0.035665159 0.343324555           Medium
*
7    1.639143 -3.27190016 0.065978233 0.326042199           Medium
ns
8    2.972476 -3.19898874 0.082216791 0.319525998           Medium
ns
9    3.472476 -3.17164695 0.089166835 0.317070627           Medium
ns
10 104.722476  2.36506432 0.600348700 0.241896012            Small
ns
11 102.055810  2.21924147 0.733814643 0.227779262            Small
ns
12  23.639143 -2.06886166 0.851019997 0.213069472            Small
ns
13  98.972476  2.05063381 0.862996463 0.211276361            Small
ns
```

14 ns	27.972476	-1.83189953	0.962332086	0.189596742	Small
15 ns	94.472476	1.80455775	0.969297135	0.186866476	Small
16 ns	29.805810	-1.73164632	0.983163203	0.179564712	Small
17 ns	88.722476	1.49012723	0.998853065	0.155170680	Small
18 ns	88.222476	1.46278545	0.999219296	0.152390234	Small
19 ns	35.139143	-1.44000063	0.999441553	0.150070426	Small
20 ns	85.972476	1.33974742	0.999892049	0.139834263	Small
21 ns	83.305810	1.19392457	0.999994399	0.124865748	Small
22 ns	81.639143	1.10278529	0.999999415	0.115466268	Small
23 ns	81.305810	1.08455743	0.999999643	0.113582561	Small
24 ns	81.139143	1.07544351	0.999999723	0.112640247	Small
25 ns	80.805810	1.05721565	0.999999835	0.110754708	Small
26 ns	79.805810	1.00253208	0.999999969	0.105090990	Small
27 ns	43.805810	-0.96607637	0.999999990	0.101309454	Small
28 ns	76.805810	0.83848138	1.000000000	0.088040495	Very small
29 ns	75.889143	0.78835477	1.000000000	0.082814440	Very small
30 ns	75.472476	0.76556995	1.000000000	0.080436674	Very small
31 ns	48.139143	-0.72911424	1.000000000	0.076629407	Very small
32 ns	58.389143	-0.16860767	1.000000000	0.017770002	Very small
33 ns	54.055810	-0.40556980	1.000000000	0.042711797	Very small
34 ns	57.139143	-0.23696213	1.000000000	0.024970213	Very small
35 ns	55.722476	-0.31443052	1.000000000	0.033125697	Very small
36 ns	65.472476	0.21873427	1.000000000	0.023050491	Very small
37 ns	58.805810	-0.14582285	1.000000000	0.015369262	Very small
38 ns	68.555810	0.38734194	1.000000000	0.040795436	Very small
39 ns	55.305810	-0.33721534	1.000000000	0.035523183	Very small
40 ns	66.889143	0.29620266	1.000000000	0.031207294	Very small

```
41  71.222476  0.53316479 1.000000000 0.056111959       Very small
ns
42  66.389143  0.26886088 1.000000000 0.028329050       Very small
ns
43  56.639143 -0.26430391 1.000000000 0.027849273       Very small
ns
44  62.805810  0.07291142 1.000000000 0.007685312       Very small
ns
45  60.972476 -0.02734178 1.000000000 0.002882065       Very small
ns


============================================================
SIGNIFICANT INTERACTION CONTRASTS
============================================================

     Treatment_pairwise Plastic_pairwise estimate       SE df  lower.CL
1   Control - Chlorella    Green - Black 85.25000 18.28703 90 23.777524
2   Control - Chlorella     Blue - Black 77.83333 18.28703 90 16.360857
3 Control - Scenedesmus     Gray - Black 69.58333 18.28703 90  8.110857
4 Control - Scenedesmus    Green - Black 66.91667 18.28703 90  5.444190
5 Control - Scenedesmus     Blue - Black 63.83333 18.28703 90  2.360857
6   Control - Chlorella     Gray - Black 63.41667 18.28703 90  1.944190
  upper.CL  t.ratio     p.value  r_effect r_interpretation significance
1 146.7225 4.661774 0.000486853 0.4410240           Medium          ***
2 139.3058 4.256204 0.002287186 0.4093351           Medium           **
3 131.0558 3.805065 0.011550811 0.3722619           Medium            *
4 128.3891 3.659242 0.018991371 0.3598751           Medium            *
5 125.3058 3.490634 0.033134803 0.3453121           Medium            *
6 124.8891 3.467850 0.035665159 0.3433246           Medium            *


============================================================
INFLUENTIAL OBSERVATIONS
============================================================

  Observation_ID Treatment Plastic WeightLoss_percent   fitted  residual
1             92 Chlorella   Black              24.14 1.928333 22.211667
2             96 Chlorella   Black              -5.60 1.928333 -7.528333
3             95 Chlorella   Black              -3.17 1.928333 -5.098333
4             93 Chlorella   Black              -3.08 1.928333 -5.008333
  studentized_residual  leverage cooks_distance abs_studentized_residual
1            28.824289 0.1666667     0.90324401                28.824289
2            -3.209990 0.1666667     0.10376251                 3.209990
3            -2.108785 0.1666667     0.04758820                 2.108785
4            -2.069750 0.1666667     0.04592289                 2.069750
  influential_Cook influential_residual influential_leverage
1             TRUE                 TRUE                FALSE
2             TRUE                 TRUE                FALSE
3             TRUE                FALSE                FALSE
4             TRUE                FALSE                FALSE
  potentially_influential
1                    TRUE
2                    TRUE
```

```
3                    TRUE
4                    TRUE


============================================================
SENSITIVITY ANALYSIS
============================================================

                Term Df Df.res   Sum Sq Sum Sq.res   F value
Pr(>F)
1          Treatment  2     90 26404.06   52720.33 22.537462
0.000000011615824
2            Plastic  5     90 10518.69   75704.33  2.501000
0.036170308538952
3  Treatment:Plastic 10     90 19110.94   45146.08  3.809821
0.000259585563536
4          Treatment  2     89 17607.60   69830.47 11.220580
0.000045115436358
5            Plastic  5     89 26522.92   55652.53  8.483136
0.000001342695310
6  Treatment:Plastic 10     89 30591.26   58336.22  4.667122
0.000024078466728
7          Treatment  2     89 27344.10   48048.70 25.324567
0.000000001966316
8            Plastic  5     89 10253.29   69102.53  2.641127
0.028351053093814
9  Treatment:Plastic 10     89 19024.18   42377.22  3.995430
0.000156462431787
10         Treatment  2     88 18013.96   63498.30 12.482450
0.000016893610310
11           Plastic  5     88 26118.94   50244.10  9.149199
0.000000488983615
12 Treatment:Plastic 10     88 29345.85   55556.18  4.648330
0.000026018344481
   partial_eta_squared significance
1            0.3337031          ***
2            0.1219940            *
3            0.2974141          ***
4            0.2013723          ***
5            0.3227597          ***
6            0.3440024          ***
7            0.3626885          ***
8            0.1292065            *
9            0.3098330          ***
10           0.2209970          ***
11           0.3420364          ***
12           0.3456437          ***
                                       Analysis   N    F_full
p_full
1                                  Full dataset 108 22.537462
0.00000001161582
2                                  Full dataset 108  2.501000
0.03617030853895
```

```
3                                  Full dataset 108  3.809821
0.00025958556354
4     Without Chlorella-Black 24.14 observation 107 22.537462
0.00000001161582
5     Without Chlorella-Black 24.14 observation 107  2.501000
0.03617030853895
6     Without Chlorella-Black 24.14 observation 107  3.809821
0.00025958556354
7     Without Control-PetWhite 2.22 observation 107 22.537462
0.00000001161582
8     Without Control-PetWhite 2.22 observation 107  2.501000
0.03617030853895
9     Without Control-PetWhite 2.22 observation 107  3.809821
0.00025958556354
10 Without both predefined extreme observations 106 22.537462
0.00000001161582
11 Without both predefined extreme observations 106  2.501000
0.03617030853895
12 Without both predefined extreme observations 106  3.809821
0.00025958556354
    eta_full         p_current conclusion_full conclusion_current
1  0.3337031 0.000000011615824     Significant        Significant
2  0.1219940 0.036170308538952     Significant        Significant
3  0.2974141 0.000259585563536     Significant        Significant
4  0.3337031 0.000045115436358     Significant        Significant
5  0.1219940 0.000001342695310     Significant        Significant
6  0.2974141 0.000024078466728     Significant        Significant
7  0.3337031 0.000000001966316     Significant        Significant
8  0.1219940 0.028351053093814     Significant        Significant
9  0.2974141 0.000156462431787     Significant        Significant
10 0.3337031 0.000016893610310     Significant        Significant
11 0.1219940 0.000000488983615     Significant        Significant
12 0.2974141 0.000026018344481     Significant        Significant
   conclusion_changed                        robustness_statement
1               FALSE Inference remained directionally consistent
2               FALSE Inference remained directionally consistent
3               FALSE Inference remained directionally consistent
4               FALSE Inference remained directionally consistent
5               FALSE Inference remained directionally consistent
6               FALSE Inference remained directionally consistent
7               FALSE Inference remained directionally consistent
8               FALSE Inference remained directionally consistent
9               FALSE Inference remained directionally consistent
10              FALSE Inference remained directionally consistent
11              FALSE Inference remained directionally consistent
12              FALSE Inference remained directionally consistent


============================================================
INTERACTION SENSITIVITY
============================================================

                       Analysis   N  F_value       p_value
partial_eta_squared
```

```
1                  Full dataset 108 3.809821 0.00025958556
0.2974141
2 Without Chlorella-Black 24.14 107 4.667122 0.00002407847
0.3440024


============================================================
END OF REPORT
============================================================
```
